# STAT3 is critical for skeletal development and bone homeostasis by regulating osteogenesis

**DOI:** 10.1038/s41467-021-27273-w

**Published:** 2021-11-25

**Authors:** Siru Zhou, Qinggang Dai, Xiangru Huang, Anting Jin, Yiling Yang, Xinyi Gong, Hongyuan Xu, Xin Gao, Lingyong Jiang

**Affiliations:** 1 Center of Craniofacial Orthodontics, Department of Oral and Cranio-maxillofacial Science, Shanghai Ninth People’s Hospital, Shanghai Jiao Tong University School of Medicine, College of Stomatology, Shanghai Jiao Tong University, National Center for Stomatology, National Clinical Research Center for Oral Disease, Shanghai Key Laboratory of Stomatology, Shanghai, 200011 China; 2 2nd Dental Center, Shanghai Ninth People’s Hospital, Shanghai Jiao Tong University School of Medicine, College of Stomatology, Shanghai Jiao Tong University, National Center for Stomatology, National Clinical Research Center for Oral Disease, Shanghai Key Laboratory of Stomatology, Shanghai, 200011 China

**Keywords:** Bone development, Musculoskeletal abnormalities, Drug development

## Abstract

Skeletal deformities are typical AD-HIES manifestations, which are mainly caused by heterozygous and loss-of-function mutations in Signal transducer and activator of transcription 3 (STAT3). However, the mechanism is still unclear and the treatment strategy is limited. Herein, we reported that the mice with *Stat3* deletion in osteoblasts, but not in osteoclasts, induced AD-HIES-like skeletal defects, including craniofacial malformation, osteoporosis, and spontaneous bone fracture. Mechanistic analyses revealed that STAT3 in cooperation with Msh homeobox 1(MSX1) drove osteoblast differentiation by promoting Distal-less homeobox 5(*Dlx5)* transcription. Furthermore, pharmacological activation of STAT3 partially rescued skeletal deformities in heterozygous knockout mice, while inhibition of STAT3 aggravated bone loss. Taken together, these data show that STAT3 is critical for modulating skeletal development and maintaining bone homeostasis through STAT3-indcued osteogenesis and suggest it may be a potential target for treatments.

## Abstract (web summary)

Autosomal dominant hyper-immunoglobulin E syndrome (AD-HIES) is associated with mutations in STAT3, and clinical manifestations include skeletal deformities. Here, the authors show that inactivation of STAT3 in osteoblast induces AD-HIES-like skeletal defects by impairing osteogenesis, and show that pharmacological STAT3 activation rescues the phenotype.

## Introduction

Autosomal dominant hyper-immunoglobulin E syndrome (AD-HIES), also known as Job’s syndrome, is a rare multisystem disease that is characterized by pathologically elevated serum immunoglobulin E (IgE) and immunological disorders, such as recurrent staphylococcal skin abscesses and cyst-forming pneumonia^1,2^. These immunological disorders of AD-HIES have received lots of attention in previous studies. Nevertheless, although a series of clinical studies have reported that skeletal deformities such as anomalies in craniofacial bone, severe osteoporosis, and recurrent minimal trauma fractures are typical and common manifestations of AD-HIES, limited research is available that addresses them^3–5^. In a clinical report from the USA and Central Europe, 89.4% (34/38) of AD-HIES patients exhibit skeletal deformities, such as characteristic facial features, and pathologic fractures^6^. The incidence of distinctive facial features of AD-HIES patients in a French study was 95% (57/60) and 90.6% (58/64) in an international study^7,8^. In China, a cohort study showed that all 17 AD-HIES patients exhibited characteristic facial features^9^. Although the bone deformities are characteristic and harmful for the health and beauty of AD-HIES patients, clinical treatment is limited. Hence, clarifying the underlying mechanisms of AD-HIES-related bone defects is important.

Signal transducer and activator of transcription (STAT3) is well known to play an important role in the development of AD-HIES^5,10–13^. As a member of the Janus kinase-signal transducers and activators of transcription (JAK-STAT) family of proteins, STAT3 responds to growth factors and multiple cytokines, and plays a critical role in regulating cell proliferation, differentiation, migration, apoptosis, and survival^14–16^. STAT3 has six domains, including the N-terminal domain (NTD), coiled-coil domain (CC), DNA-binding domain (DBD), linker domain (linker), the Src homology 2 (SH2) domain, and the transactivation domain (TD). Previous research has shown that the STAT3 mutations in AD-HIES patients are located in the DNA-binding domain (DBD), the Src homology 2 (SH2) domain, and the transactivation domain (TAD), and these mutations induced STAT3 loss-of-function mainly through the mechanism of negative dominance^9,17^. The previous studies have focused on the immune system abnormalities induced by the inactivation of STAT3 in AD-HIES patients^18–22^, but the bone abnormalities have been less studied^23–25^.

Skeletal development and bone homeostasis depend on the balance of the resorption of old or damaged bone by osteoclasts and formation of new bone by osteoblasts. Imbalance of this tightly coupled process may induce diseases such as osteoporosis and spontaneous fracture^26^. Bone deformity and decreased bone mass can be the result of decreased bone formation, increased bone resorption, or a combination of both effects. Several lines of data suggested that the osteoporosis induced by STAT3 inactivation may be relevant to incremented osteoclast activity^27–29^. Therefore, bisphosphonates, which could inhibit osteoclast activity and bone resorption, was be leveraged to treat secondary osteoporosis that caused by STAT3 mutations^30^. However, clinical studies have shown that although bisphosphonates increase bone mineral density, and bisphosphonates do not effectively reduce the incidence of pathological fractures in patients^31,32^. Furthermore, the treatment strategy of anomalies in the craniofacial bone of AD-HIES patients has been rarely reported^33^. To solve this clinical difficulty and develop new treatment insights and methods, the pathogenesis of bone development malformation caused by STAT3 inactivation and the underlying mechanisms must be furthermore elucidated.

Here we show the evidence that STAT3 deficiency in bone marrow mesenchymal stem cells (BMSCs) or pre-osteoblasts, but not in osteoclasts, induces craniofacial deformity, osteoporosis, and spontaneous bone fractures, resembling the cranial bone phenotypes of AD-HIES patients. Furthermore, the bone defects in osteoblast-specific *Stat3-*deficient mice indicate that the impaired bone formation but not the increased bone resorption is the contributor for this phenotype, which may provide potential insights for the treatment of bone abnormalities in AD-HIES patients.

## Results

### Deletion of *Stat3* in osteoclasts did not induce AD-HIES-like skeletal deformity

**Fig. 1:**
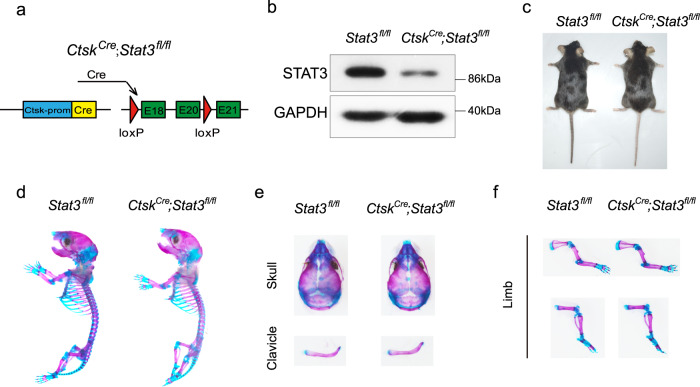
Deletion of *Stat3* in osteoclasts did not cause AD-HIES-like bone deformities. **a** Illustration of *Stat3* deletion in *Ctsk*-expressing osteoclasts. **b** Western blotting of STAT3 in BMMSs from 4-week-old male *Stat3*^*fl/fl*^ and *Ctsk*^*Cre*^*;Stat3*^*fl/fl*^ mice cultured with M-CSF and RANKL for 7 days. The experiment was performed twice to similar results. **c** Representative views of 8-week-old male *Stat3*^*fl/fl*^ and *Ctsk*^*Cre*^*;Stat3*^*fl/fl*^ mice. **d**–**f** Skeletal preparations from male *Stat3*^*fl/fl*^ and *Ctsk*^*Cre*^*;Stat3*^*fl/fl*^ newborns were double-stained with alcian blue and alizarin red S. Source data are provided in the Source data file.

Bone development and remodeling depend on the balance between bone resorption by osteoclasts and bone formation by osteoblasts. If this balance is broken, both developmental and metabolic bone diseases could be induced. Previous reports have indicated that AD-HIES-related osteoporosis may be related to abnormal bone resorption^27,28^. Hence, we first generated an osteoclast-specific *Stat3* knockout mouse model (*Ctsk*^*Cre*^;*Stat3*^*fl/fl*^ mice) by crossing *Stat3*^*fl/fl*^ mice with cathepsin K-Cre (*Ctsk*^*Cre*^) mice, a line in which Cre expression is primarily restricted to osteoclasts (Fig. 1a). Decreased expression of STAT3 in bone marrow macrophages (BMMs) from *Ctsk*^*Cre*^;*Stat3*^*fl/fl*^ mice was confirmed by western blotting (Fig. 1b). The physical size of the *Ctsk*^*Cre*^;*Stat3*^*fl/fl*^ mice was similar to their *Stat3*^*fl/fl*^ littermates (Fig. 1c). Alcian blue and alizarin red S staining did not show any developmental bone deformity in the *Ctsk*^*Cre*^;*Stat3*^*fl/fl*^ mice (Fig. 1d–f). Furthermore, our previous study indicated that *Ctsk*^*Cre*^;*Stat3*^*fl/fl*^ mice exhibited increased bone mass^34^. All these results validated that deleting *Stat3* in osteoclasts could not induce the AD-HIES-like skeletal deformities.

### Deletion of *Stat3* in pre-osteoblasts induced AD-HIES-related skeletal deformities

**Fig. 2:**
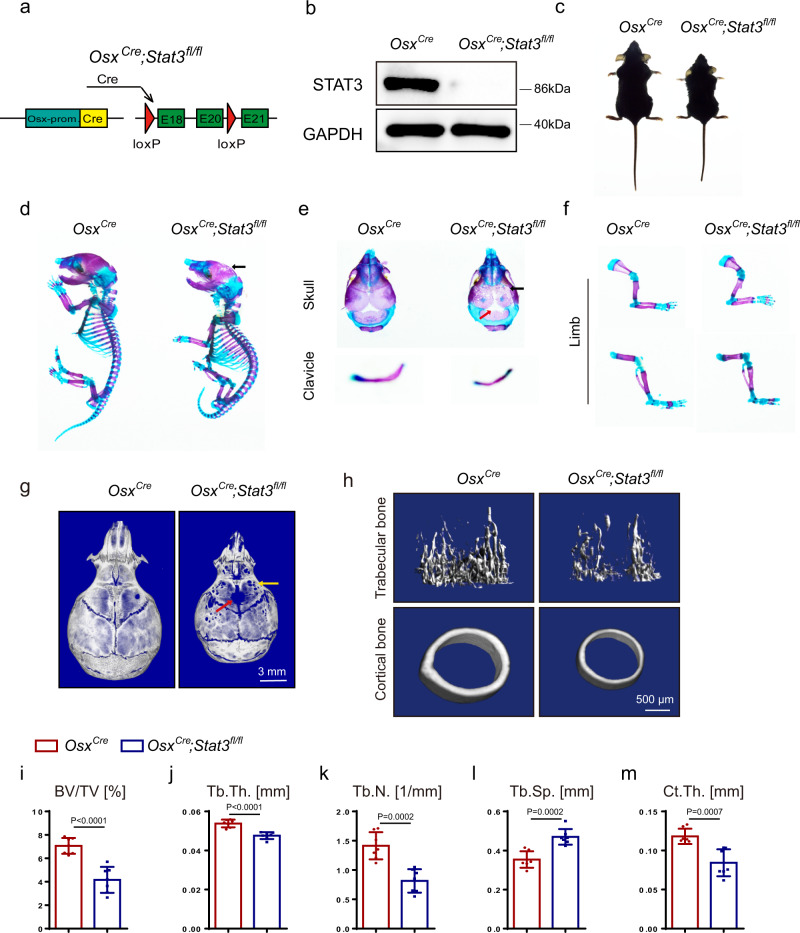
Deletion of *Stat3* in pre-osteoblasts induced skeletal deformities. **a** Illustration of *Stat3* deletion in *Osx*-expressing pre-osteoblasts. **b** Western blotting of STAT3 in BMSCs from 4-week-old male *Stat3*^*fl/fl*^ and *Osx*^*Cre*^*;Stat3*^*fl/fl*^ mice cultured in osteogenic medium for 7 days. The experiment was performed twice to similar results. **c** Representative views of 8-week-old male *Osx*^*Cre*^ and *Osx*^*Cre*^*;Stat3*^*fl/fl*^ mice. **d**–**f** Skeletal preparations from male *Osx*^*Cre*^ and *Osx*^*Cre*^*;Stat3*^*fl/fl*^ newborns were double-stained with alcian blue and alizarin red S. Arrows indicate calvarial lesions. **g** Microcomputed tomography (micro-CT) images of skulls from 4-week-old male *Osx*^*Cre*^ and *Osx*^*Cre*^*;Stat3*^*fl/fl*^ mice. Arrows indicate calvarial lesions. **h** Micro-CT images of distal femurs from 4-week-old male *Osx*^*Cre*^ and *Osx*^*Cre*^*;Stat3*^*fl/fl*^ mice. **i**–**m** Quantitative parameters of micro-CT, including bone volume per tissue volume (BV/TV), trabecular thickness (Tb.Th.), trabecular number (Tb.N.), trabecular space (Tb.Sp.), and cortical thickness (Ct.Th). *n* = 7. Two-tailed Student’s *t* test. Data represent the mean ± s.d. Source data are provided in the Source data file.

To further determine whether the inactivation of STAT3 in osteoblast lineage cells was the cause of the AD-HIES-related skeletal deformities, we generated a pre-osteoblast-specific *Stat3* deletion mouse model (*Osx*^*Cre*^;*Stat3*^*fl/fl*^ mice) by crossing *Stat3*^*fl/fl*^ mice with Osterix-Cre (*Osx*^*Cre*^) mice, a line in which Cre expression is primarily restricted to osteoblast precursors (Fig. 2a). We made a comparison between *Osx*^*Cre*^;*Stat3*^*fl/fl*^ mice and Osx-Cre control (*Osx*^*Cre*^) littermates that were both in the context of Osx-Cre to exclude the effects of *Osx-Cre*^35,36^. Western blot confirmed the deletion of STAT3 in BMSCs of the *Osx*^*Cre*^;*Stat3*^*fl/fl*^ mice (Fig. 2b). The physical size analysis showed that 8-week-old *Osx*^*Cre*^;*Stat3*^*fl/fl*^ mice were dwarfed relative to their control littermates (Fig. 2c). Alcian blue and alizarin red S staining displayed defects in the skeleton of the *Osx*^*Cre*^;*Stat3*^*fl/fl*^ newborns, including hypomineralization of the cranial bones and hypoplasia of the clavicle (Fig. 2d–f). Further, micro-CT was used to analyze the skulls of 4-week-old *Osx*^*Cre*^;*Stat3*^*fl/fl*^ mice and control littermates. The skulls of the *Osx*^*Cre*^;*Stat3*^*fl/fl*^ mice exhibited not only morphological deformity, including craniosynostosis and circular shape, but also hypo-ossification, indicated by obvious porosity when compared with the *Osx*^*Cre*^ littermates (Fig. 2g). To further determine the function of STAT3 in the skeletal system, we utilized micro-CT to analyze the skeletal elements isolated from the *Osx*^*Cre*^;*Stat3*^*fl/fl*^ mice with corresponding control littermate *Osx*^*Cre*^ mice. As shown in Fig. 2h–m, 4-week-old *Osx*^*Cre*^;*Stat3*^*fl/fl*^ mice had osteoporosis with a reduced bone volume per tissue volume (BV/TV) in the femoral trabecular bone relative to the age-matched control littermates. Further analysis showed that the *Osx*^*Cre*^;*Stat3*^*fl/fl*^ mice displayed a decrease in trabecular thickness (Tb.Th.) and trabecular number (Tb.N.) but an increase in trabecular separation (Tb.Sp.). Also, *Osx*^*Cre*^;*Stat3*^*fl/fl*^ mice displayed a dramatic reduction in the cortical thickness (Ct.Th.) compared with the control *Osx*^*Cre*^ littermates. Taken together, the deletion of *Stat3* in pre-osteoblasts induced AD-HIES-related skeletal deformities, such as craniofacial deformities and osteoporosis.

### Ablation of *Stat3* in BMSCs resulted in skeletal deformities

**Fig. 3:**
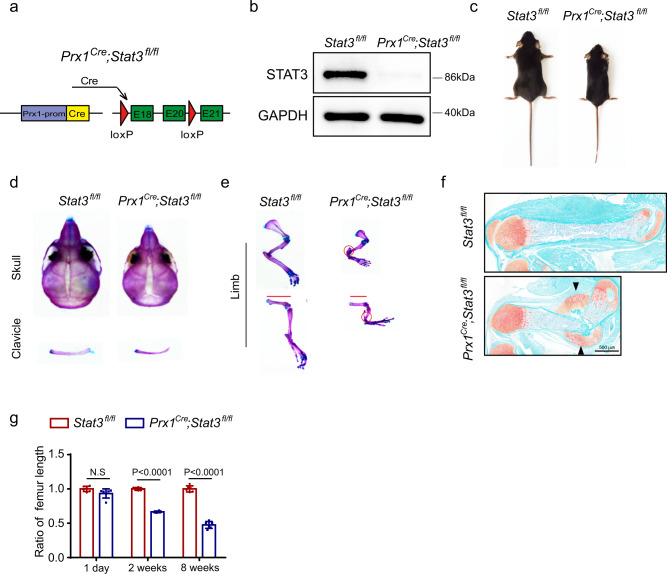
Ablation of *Stat3* in BMSCs induced bone defects in mice. **a** Illustration of *Stat3* deletion in Prx1-expressing bone mesenchymal stem cells. **b** Western blotting of STAT3 in BMSCs from 4-week-old male *Stat3*^*fl/fl*^ and *Prx1*^*Cre*^*;Stat3*^*fl/fl*^ mice cultured in osteogenic medium for 7 days. The experiment was performed twice to similar results. **c** Representative views of 8-week-old male *Stat3*^*fl/fl*^ and *Prx1*^*Cre*^*;Stat3*^*fl/fl*^ mice. **d**, **e** Skeletal preparations from 2-week-old male *Stat3*^*fl/fl*^ and *Prx1*^*Cre*^*;Stat3*^*fl/fl*^ were double-stained with alcian blue and alizarin red S. Red circles represent bone fracture. **f** Safranin O stain of femurs to show bone fractures in 1-week-old *Prx1*^*Cre*^*;Stat3*^*fl/fl*^ mice. Black arrows indicate bony callus. **g** Statistical analysis of femur length from different ages of *Stat3*^*fl/fl*^ and *Prx1*^*Cre*^*;Stat3*^*fl/fl*^ mice. *n* = 6. Two-tailed Student’s *t* test. Data represent the mean ± s.d. Source data are provided in the Source data file.

To further explore the role of STAT3 in the commitment of BMSCs to an osteoblast fate, we generated osteoblast lineage-specific *Stat3*-deficient mice (*Prx1*^*Cre*^;*Stat3*^*fl/fl*^ mice) by crossing *Stat3*^*fl/fl*^ mice with Prx1-Cre (*Prx1*^*Cre*^) mice (Fig. 3a). The latter is a transgenic line that has Cre expression in a subset of craniofacial mesenchyme and throughout the early limb bud mesenchyme^37^. Western blot analysis verified that STAT3 decremented in BMSCs (Fig. 3b). The 8-week-old *Prx1*^*Cre*^;*Stat3*^*fl/fl*^ mice were dwarfed relative to their control littermates (*Stat3*^*fl/fl*^) (Fig. 3c, g). Alcian blue and alizarin red S staining showed defects and malformation in the skeleton of newborns, 2- and 8-week-old *Prx1*^*Cre*^;*Stat3*^*fl/fl*^ mice compared with *Stat3*^*fl/fl*^ control littermates (Fig. 3d, e, Supplementary Fig. 1). Notably, *Prx1*^*Cre*^;*Stat3*^*fl/fl*^ mice had spontaneous fractures (Fig. 3e, f, Supplementary Fig. 1b, d), similar to the spontaneous bone fractures observed in AD-HIES patients. Thus, both *Osx*^*Cre*^;*Stat3*^*fl/fl*^ and *Prx1*^*Cre*^;*Stat3*^*fl/fl*^ mice, but not *Ctsk*^*Cre*^;*Stat3*^*fl/fl*^ mice, reproduced the bone phenotype of AD-HIES patients, including craniofacial dysplasia, osteoporosis, and spontaneous bone fractures. This indicated that inactivation of STAT3 in osteoblasts, but not osteoclasts, induced the AD-HIES-related bone defects.

### Ablation of *Stat3* in pre-osteoblasts impaired osteogenesis by inhibiting osteoblast differentiation

**Fig. 4:**
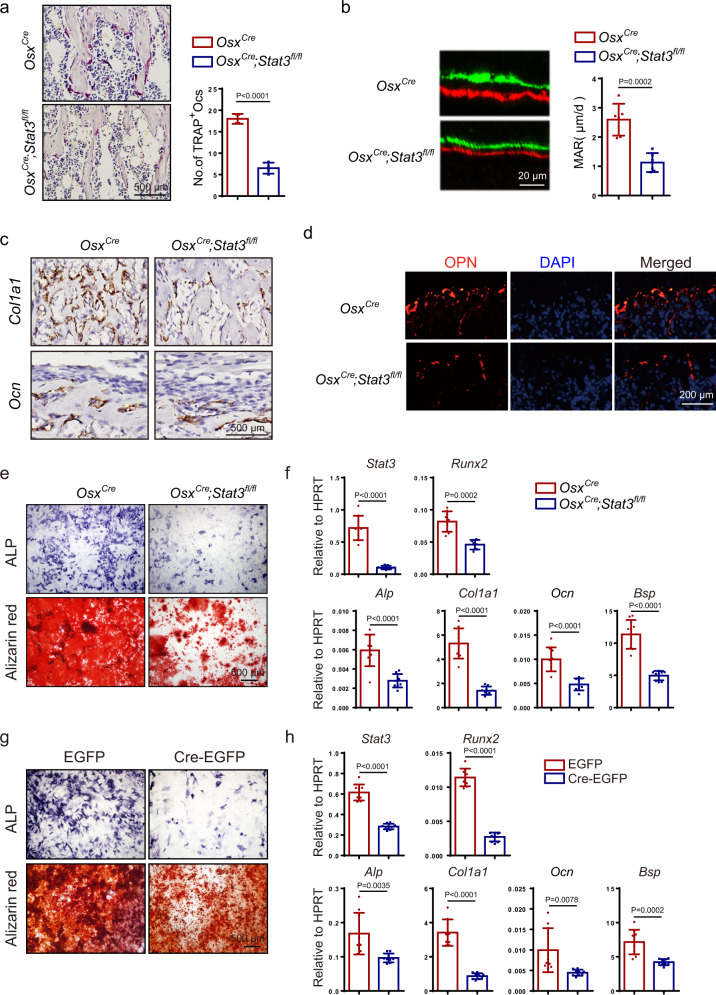
STAT3-driven osteoblast differentiation and bone formation. **a** TRAP staining of femurs from 4-week-old male *Osx*^*Cre*^ and *Osx*^*Cre*^*;Stat3*^*fl/fl*^ mice and analysis of the number of TRAP^+^-multinucleated osteoclasts. *n* = 4. **b** Representative images of calcein-alizarin red S double labeling of femurs from 4-week-old male *Osx*^*Cre*^ and *Osx*^*Cre*^*;Stat3*^*fl/fl*^ mice and quantitative parameters mineral apposition rate (MAR). *n* = 6. **c** In situ hybridization for *Col1α1* and *Ocn* in femurs from 1-week-old male *Osx*^*Cre*^ and *Osx*^*Cre*^*;Stat3*^*fl/fl*^ littermates. **d** Immunofluorescence staining for OPN in distal femurs from 1-week-old male *Osx*^*Cre*^ and *Osx*^*Cre*^*;Stat3*^*fl/fl*^ mice. **e** ALP staining and alizarin red S staining of BMSCs after culture in osteogenic medium for 7 days and 14 days separately. **f** The relative mRNA levels of *Stat3, Runx2, Alp, Col1α1, Ocn*, and *Bsp* in BMSCs after culture in osteogenic medium for 14 days. *n* = 9. **g** BMSCs from 4-week-old male *Stat3*^*fl/fl*^ mice were infected with adenovirus expressing EGFP or Cre-EGFP for 4 h then induced by osteogenic medium; ALP staining and alizarin red S staining of BMSCs after culture in osteogenic medium for 7 days and 14 days separately. **h** The relative mRNA levels of *Stat3, Runx2, Alp, Col1α1, Ocn*, and *Bsp* were quantified after 14 days of culture in osteogenic medium. *n* = 9. Two-tailed Student’s *t* test. Data represent the mean ± s.d. Source data are provided in the Source data file.

Throughout life, bone is continuously modeled and remodeled by the functions of osteoblasts and osteoclasts^38^. Bone deformity and decreased bone mass can be the result of decreased bone formation, increased bone resorption, or a combination of both effects. To understand how *Stat3* deletion caused the bone abnormity, we first analyzed femurs from 4-week-old mice for the presence of tartrate-resistant acid phosphatase (TRAP)-positive osteoclasts. Compared with *Osx*^*Cre*^ littermates, *Osx*^*Cre*^;*Stat3*^*fl/fl*^ mice displayed a decrease in the number of TRAP-positive osteoclasts, excluding the possibility that the bone loss in osteoblast-specific *Stat3*-deficient mice was induced by an increase in bone resorption (Fig. 4a). Next, we analyzed the bone formation ability in *Osx*^*Cre*^;*Stat3*^*fl/fl*^ mice using calcein-alizarin red S double labeling. We found that bone formation activity was lower in the *Osx*^*Cre*^;*Stat3*^*fl/fl*^ mice than the *Osx*^*Cre*^ mice (Fig. 4b). Consistent with the reduced ossification and bone formation rate, the in situ hybridization analysis showed that the expression of the osteoblast-specific marker genes collagen1α1 (*Col1α1*) and osteocalcin (*Ocn*) were reduced in osteoblasts in vivo from *Osx*^*Cre*^;*Stat3*^*fl/fl*^ mice (Fig. 4c). Consistent with this, the number of the mature osteopontin (OPN)-positive osteoblasts was reduced in the *Osx*^*Cre*^;*Stat3*^*fl/fl*^ mice (Fig. 4d). All these results suggested that the bone deformities and decreased bone mass of the *Osx*^*Cre*^;*Stat3*^*fl/fl*^ mice were resulted from decreased bone formation rather than increased bone resorption.

Osteoblasts are derived from BMSCs^38–40^. Hence, we wondered whether *Stat3* deficiency would affect the osteogenic potential of BMSCs. Primary BMSCs were isolated from the bone marrow of *Osx*^*Cre*^;*Stat3*^*fl/fl*^ mice and control littermates. *Osx*^*Cre*^;*Stat3*^*fl/fl*^ BMSCs displayed impaired osteoblast differentiation ability as indicated by the markedly decreased alkaline phosphatase (ALP) activity and lower mineralization by alizarin red S staining (Fig. 4e). This was consistent with a decrease in the mRNA level of osteogenic genes, such as *Runx2, Alp, Col1a1, Ocn*, and *Bsp* (Fig. 4f). Since *Osx*^*Cre*^;*Stat3*^*fl/fl*^ mice had grossly deformed bones, to exclude the developmental differences, we infected *Stat3*^*fl/fl*^ primary BMSCs with adenovirus expressing either Cre-EGFP targeting *Stat3* or control EGFP. Consistent with BMSCs from *Osx*^*Cre*^;*Stat3*^*fl/fl*^ mice, the deletion of *Stat3* in BMSCs in vitro also resulted in decreased osteoblast differentiation, as indicated by decreased ALP activity and mineralization, as well as deregulated expression of osteogenic marker genes (Fig. 4g–h). Collectively, these results demonstrated that STAT3 was required for osteoblast differentiation in a cell-autonomous manner.

### STAT3 activity was required for osteogenic gene transcription

**Fig. 5:**
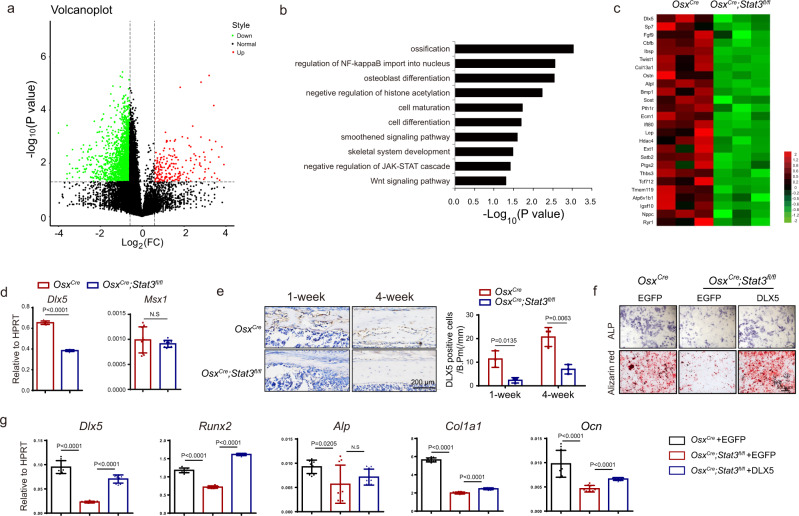
STAT3 regulated the gene network of osteoblast differentiation. **a** Total RNA was isolated from calvaria bone of male *Osx*^*Cre*^ and *Osx*^*Cre*^*;Stat3*^*fl/fl*^ newborns, followed by RNA sequencing analysis. Kolmogorov–Smirnov (K–S) test was used for testing the correlation between the *Osx*^*Cre*^ set and *Osx*^*Cre*^*;Stat3*^*fl/fl*^ set. Volcano plot displays global gene expression in the *Osx*^*Cre*^ and *Osx*^*Cre*^*;Stat3*^*fl/fl*^ sets. Two-sided test. Green represents downregulated genes, red represents upregulated genes. **b** Gene ontology (GO) enrichment analysis of downregulated genes (>1.5-fold) in the *Osx*^*Cre*^*;Stat3*^*fl/fl*^ set. **c** Heatmap analysis of osteoblast-related genes in the *Osx*^*Cre*^ and *Osx*^*Cre*^*;Stat3*^*fl/fl*^ sets. **d** The relative mRNA levels of *Dlx5* and *Msx1* from the calvaria of *Osx*^*Cre*^ and *Osx*^*Cre*^*;Stat3*^*fl/fl*^ newborns were quantified by qPCR. *n* = 9 **e** Immunohistochemistry for DLX5 in femurs from 1-week-old and 4-week-old *Osx*^*Cre*^ and *Osx*^*Cre*^*;Stat3*^*fl/fl*^ littermates. DLX5-positive cells were counted. *n* = 3. **f** BMSCs from 4-week-old male *Osx*^*Cre*^ and *Osx*^*Cre*^*;Stat3*^*fl/fl*^ mice were infected with lentivirus expressing EGFP or DLX5 for 24 h then induced by osteogenic medium; ALP staining and alizarin red S staining of BMSCs after culture in osteogenic medium for 7 days and 14 days separately. **g** The relative mRNA levels of *Dlx5, Runx2, Alp, Col1α1,* and *Ocn* were quantified after 14 days of culture in osteogenic medium. *n* = 9. Two-tailed Student’s *t* test. Data represent the mean ± s.d. Source data are provided in the Source data file.

To analyze the global effects of deleting *Stat3* on the gene expression in osteoblasts, we performed RNA-seq with total RNA extracted from the calvarias of newborn *Osx*^*Cre*^;*Stat3*^*fl/fl*^ and *Osx*^*Cre*^ littermates. Overall, 2459 genes were downregulated >1.5-fold in the *Osx*^*Cre*^;*Stat3*^*fl/fl*^ set compared with the corresponding control, and 231 genes were upregulated >1.5-fold in the *Osx*^*Cre*^;*Stat3*^*fl/fl*^ set compared with the control (Fig. 5a). Then, we performed a gene ontology (GO) analysis on the set of 1.5-fold significantly downregulated genes from the *Osx*^*Cre*^;*Stat3*^*fl/fl*^ samples (Fig. 5b). The results showed that these genes were enriched for associations with ossification, osteoblast differentiation, and skeletal system development, which were consistent with the skeletal deformities in the *Osx*^*Cre*^;*Stat3*^*fl/fl*^ mice, such as delayed closure of the fontanelles and decreased bone density. A heatmap further revealed that osteoblast-related gene expression, such as *Dlx5, Sp7, Fgf9, Cbfb, Ibsp*, and *Pth1r* was downregulated (Fig. 5c).

Osteoblast differentiation is achieved by activating a transcriptional network in which *Dlx5, Runx2*, and *Sp7* have essential roles^41^. Among them, *Dlx5*^*−/−*^ mice present with craniofacial abnormalities, delayed ossification of the roof of the skull, and abnormal osteogenesis^42^, which was similar to the craniofacial deformities of the *Osx*^*Cre*^;*Stat3*^*fl/fl*^ mice. Hence, we firstly confirmed *Dlx5* downregulation expression in the calvaria from *Osx*^*Cre*^;*Stat3*^*fl/fl*^ and *Osx*^*Cre*^ newborns by real-time PCR (Fig. 5d), which was consisted with the results in the *Stat3* deleted BMSCs in vitro (Supplementary Fig. 2a). Then we performed immunohistochemistry for *Dlx5* in the distal femurs from 1- and 4-week-old *Osx*^*Cre*^;*Stat3*^*fl/fl*^ mice. As shown in Fig. 5E, DLX5-positive osteoblasts were decreased in the *Osx*^*Cre*^;*Stat3*^*fl/fl*^ mice compared with that in the control group. To further verify whether decreased DLX5 expression contributed to the impaired osteoblast differentiation and ossification caused by STAT3 deficiency, we isolated BMSCs from *Osx*^*Cre*^;*Stat3*^*fl/fl*^ mice and control littermates. Then, DLX5 was overexpressed in *Osx*^*Cre*^;*Stat3*^*fl/fl*^ BMSCs using lentivirus (Lv-DLX5) (Fig. 5g). As shown in Fig. 5f, the impaired osteoblast differentiation and ossification of the *Osx*^*Cre*^;*Stat3*^*fl/fl*^ BMSCs were partially restored by DLX5 overexpression, as determined by the increased ALP activity and calcified nodules. This was consistent with the increased expression of the osteoblast marker genes, such as *Runx2, Alp, Col1a1*, and *Ocn* (Fig. 5g). All these results implied that STAT3 participated in osteoblast differentiation and ossification by controlling DLX5 expression.

**Fig. 6:**
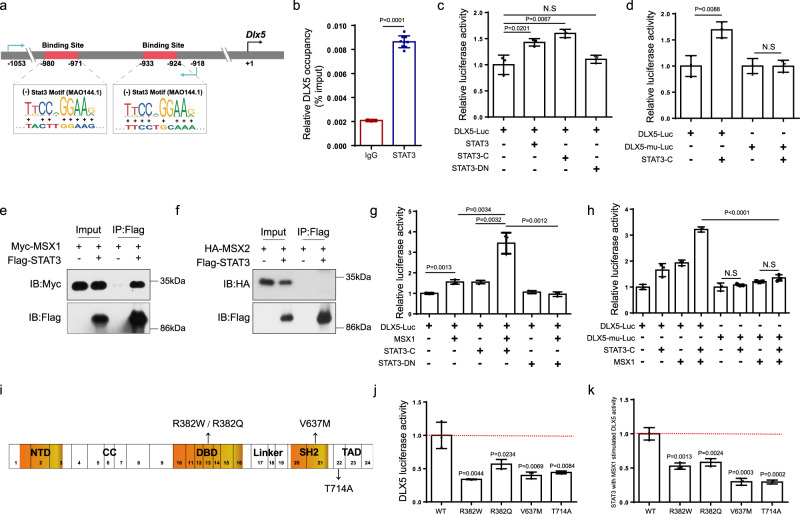
STAT3 regulated *Dlx5* transcription by promoting the activity of its promoter. **a** Illustration of predicted STAT3 binding sites on the *Dlx5* promoter. **b** ChIP assay for occupation of STAT3 on the promoter of *Dlx5* in the C3H10 cell line. IgG *n* = 3, STAT3 *n* = 9. **c** Effect of STAT3, constitutively-active STAT3 (STAT3-C) and dominant-negative mutation of STAT3 (STAT3-DN) on *Dlx5* promoter activity. *Dlx5* promoter-driven luciferase reporter was co-transfected with STAT3, STAT3-C, and STAT3-DN expressing plasmid separately in the 293T cell line. After 48 h, the luciferase activity was performed in cell extract supernatants. *n* = 3. **d** Effects of potential STAT3 binding site mutations on the activity of *Dlx5*. STAT3 was co-transfected with the luciferase reporter driven by the *Dlx5* promoter (DLX5-Luc) or mutant *Dlx5* promoter (*Dlx5* promoter with deletion of the two predicted STAT3 binding sites, DLX5-mu-Luc) separately in the 293T cell line, followed by luciferase activity analysis. *n* = 3. **e** Co-IP analysis of STAT3 and MSX1. Myc-MSX1 expressing plasmid was co-transfected with or without Flag-STAT3 in the 293T cell line. After 48 h, the whole-cell lysate was used for immunoprecipitation and then immunoblotted with the indicated antibodies. **f** Co-IP of STAT3 with MSX2. The Co-IP was repeated twice to similar results. **g** Effect of STAT3 activity on the synergistic promotion of STAT3 and MSX1 on *Dlx5* promoter activity. MSX1 and DLX5-Luc were co-transfected with STAT-C or STAT3-DN into the 293T cell line followed by luciferase activity analysis. *n* = 3. **h** Effect of STAT3 binding sites on the synergistic promotion of STAT3 and MSX1 on the *Dlx5* promoter activity. STAT3-C and MSX1 were co-transfected with the luciferase reporter driven by the *Dlx5* promoter or mutant *Dlx*5 promoter into the 293T cell line, followed by luciferase activity analysis. *n* = 3. **i** Four mutants (R382W, R382Q, V637M, and T714A) were shown on the STAT3 domains. **j** Effect of STAT3 wild type (WT) and four mutants (R382W, R382Q, V637M, and T714A) on *Dlx5* promoter activity. *n* = 3. **k** Effect of various STAT3 mutants activity on the synergistic promotion of STAT3 and MSX1 on *Dlx5* promoter activity. *n* = 3. Two-tailed Student’s *t* test. Data represent the mean ± s.d. Source data are provided in the Source data file.

Next, we investigated the mechanisms by which STAT3 regulates DLX5 expression. We first analyzed the promoter of *Dlx5* and found two potential STAT3 binding sites (Fig. 6a), which suggested that STAT3 might directly regulate the transcription of *Dlx5*. Our data conformed that STAT3 was bound to the promoter of *Dlx5* when the antibody against STAT3 was utilized for immunoprecipitation comparing with control IgG (Fig. 6b), advising that STAT3 bound directly to *Dlx5* promoter. Then, we cloned the promoter of *Dlx5* into the PGL3-basic vector to drive the expression of the luciferase reporter. We transfected a vector of STAT3, a constitutively-active STAT3 (STAT3-C, a gift from Dr. Feng^43^), and a dominant-negative STAT3 mutation (STAT3-DN, from Dr. Feng^43^) separately with *Dlx5* promoter-driven luciferase reporter. As illustrated in Fig. 6c, both STAT3 and the constitutively-active STAT3 could promote the activity of the *Dlx5* promoter. However, the dominant-negative mutation of STAT3 barely affected the activity of the *Dlx5* promoter, indicating that STAT3 activity is critical to promote the transcription of *Dlx5*. Furthermore, we constructed a mutant *Dlx5* promoter in which the two predicted STAT3 binding sites were deleted in the PGL3-basic vector (DLX5-mu). As shown in Fig. 6d, mutations in the STAT3 binding sites abrogated the activity of STAT3 on the *Dlx5* promoter activity, indicating that the two STAT3 binding sites on the *Dlx5* promoter were essential for its transcription. All these results demonstrated that STAT3 drove *Dlx5* transcription by directly regulating its promoter activity.

Craniofacial development depends on the regulation of specific transcription networks, which include the mammalian *Msx* homeobox genes (*Msx1* and *Msx2*) and distal-less (*Dlx*) gene family^44^. Previous studies have shown that *Msx1* and *Dlx5* function synergistically to regulate craniofacial development and that *Msx* is crucial for *Dlx5* transcription through binding to its promoter^45^. However, whether STAT3 participates in the functional complex between *Msx* and *Dlx5* has remained unknown. We transfected 293T cells with Myc-MSX1 and HA-MSX2 plasmids separately and with Flag-STAT3 and performed a co-immunoprecipitation (Co-IP) assay, which revealed that STAT3 physically associated with MSX1 (Fig. 6e) but not MSX2 (Fig. 6f). The transcription of *Msx1* was unchanged due to *Stat3* deletion in vivo (Fig. 5d) and in vitro (Supplementary Fig. 2a). Next, we co-transfected STAT3-C and STAT3-DN vectors separately with MSX1 vectors into 293T cells and examined their effect on the *Dlx5* reporter activity. We found that MSX1 increased the *Dlx5* promoter activity, and that STAT3-C, but not STAT3-DN, could further promote MSX1-driven *Dlx5* promoter activity (Fig. 6g). Furthermore, mutations in the STAT3 binding sites blocked the synergistic effects of STAT3 and MSX1 on the *Dlx5* promoter activity (Fig. 6h). These data inferred that STAT3 cooperated with MSX1 to drive *Dlx5* transcription and that this was dependent on the STAT3 activity.

Former study has reported that most of the HIES mutations were located in the DBD (42%) and SH2 domain (40%), of which the most frequent mutations are R382Q/W of exon 13 and V637M of exon 21, respectively^46^. In healthy individuals, alternative splicing of exon 23 leads to two major STAT3 isoforms: STAT3 alpha and STAT3 beta. The splice site mutation on exon 22 such as T714A may cause frame shift, which could possibly affect tyrosine and serine phosporylation sites of STAT3 alpha^7^. To analyze if these STAT3 mutations in AD-HIES patients could affect *Dlx5* promoter activity, we synthesized STAT3 WT (NM_001369512.1), R382W, R382Q, V637M, T714A mutation plasmids and tested their effect on the transcriptional activity of *Dlx5* promoter. As demonstrated in Fig. 6j, the *Dlx5* promoter activity in STAT3 mutation groups decremented comparing with WT group. Although STAT3 WT combined with MSX1 could synergistically promote *Dlx5* promoter activity, however, this synergistic effect with MSX1 was observed in STAT3 mutant group (Fig. 6k). These data implied that mutants found in AD-HIES patients may affect *Dlx5* transcription.

### Inducible osteoblastic deletion of *Stat3* impaired bone remodeling and reduced bone mass in adult mice

**Fig. 7:**
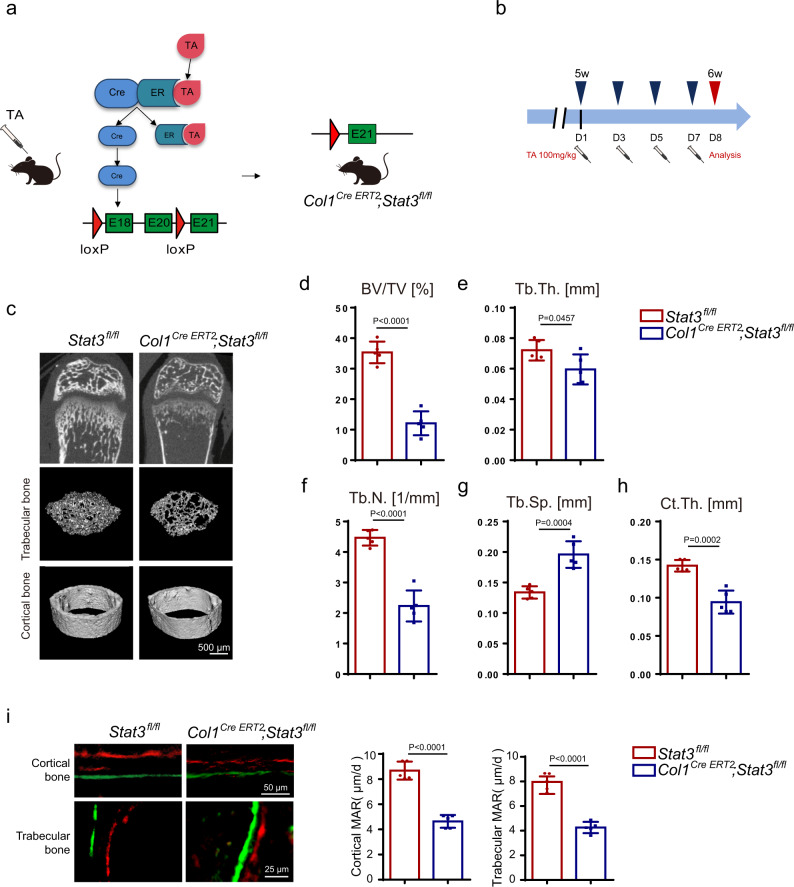
Inducible osteoblastic deletion of *Stat3* impaired bone formation and reduced bone mass in adult mice. **a** Illustration of inducible *Stat3* deletion in *Col1a1*-expressing osteoblast lineage cells (specifically, the mouse icon is bought from https://www.shutterstock.com). When *Col1*^*Cre ERT2*^*;Stat3*^*fl/fl*^ mice were injected with tamoxifen, Cre translocated to the nucleus and exon 18 to 20 domain were deleted (TA: tamoxifen). **b** Scheme of the experiment: 5-week-old male *Stat3*^*fl/fl*^ and *Col1*^*Cre ERT2*^*;Stat3*^*fl/fl*^ mice were injected with tamoxifen (100 mg/kg) for 7 days and euthanized for analysis. **c** Micro-CT images of the distal femurs from 6-week-old *Stat3*^*fl/fl*^ and *Col1*^*Cre ERT2*^*;Stat3*^*fl/fl*^ mice. **d**–**h** Quantitative parameters of micro-CT, including BV/TV, Tb.Th., Tb.N., Tb.Sp., and Ct.Th. *n* = 5. **i** Images of calcein-alizarin red S double labeling of femurs from 6-week-old *Stat3*^*fl/fl*^ and *Col1*^*Cre ERT2*^*;Stat3*^*fl/fl*^ mice and quantitative parameters of MAR. *n* = 5. Two-tailed Student’s *t* test. Data represent the mean ± s.d. Source data are provided in the Source data file.

In the above experiments using the *Prx1*^*Cre*^ and *Osx*^*Cre*^ mice, we revealed the role of STAT3 in osteoblast and bone development. We then wondered whether STAT3 played a role in bone remodeling and bone metabolism in adult mice. We used tamoxifen-inducible osteoblast lineage cells expressing Cre (*Col1*^*Cre ERT2*^) to induce deletion of *Stat3* in 5-week-old mice for 7 days (Fig. 7a, b). To determine the in vivo effects of *Stat3* deletion in this system, we preformed micro-CT analysis on the femurs from *Col1*^*Cre ERT2*^;*Stat3*^*fl/fl*^ mice and the corresponding control *Stat3*^*fl/fl*^ mice. We found that the trabecular bone in the *Col1*^*Cre ERT2*^;*Stat3*^*fl/fl*^ mice had dramatic osteoporosis compared to the *Stat3*^*fl/fl*^ mice, as indicated by decreased BV/TV, Tb.Th., Tb.N., and increased Tb.Sp. (Fig. 7c–g). Meanwhile, Ct.Th. was also decreased in the *Col1*^*Cre ERT2*^;*Stat3*^*fl/fl*^ mice (Fig. 7c, h). Consistent with our previous findings in the *Osx*^*Cre*^;*Stat3*^*fl/fl*^ mice, bone formation in the *Col1*^*Cre ERT2*^;*Stat3*^*fl/fl*^ mice was decreased compared with *Stat3*^*fl/fl*^ mice (Fig. 7i), which was accompanied with decreased bone resorption (Supplementary Fig. 3a). The data illustrated that STAT3 was critical for modulating bone development and maintaining bone homeostasis, and the STAT3 activity may function as a target to regulate bone metabolism.

### STAT3 may act as a suitable pharmacological target to regulate bone homeostasis

STAT3 has been considered as a potential target in a series of diseases, including cancer and immune disorders; therefore, we wondered whether STAT3 could be a suitable pharmacological target to regulate bone metabolism and bone homeostasis.

**Fig. 8:**
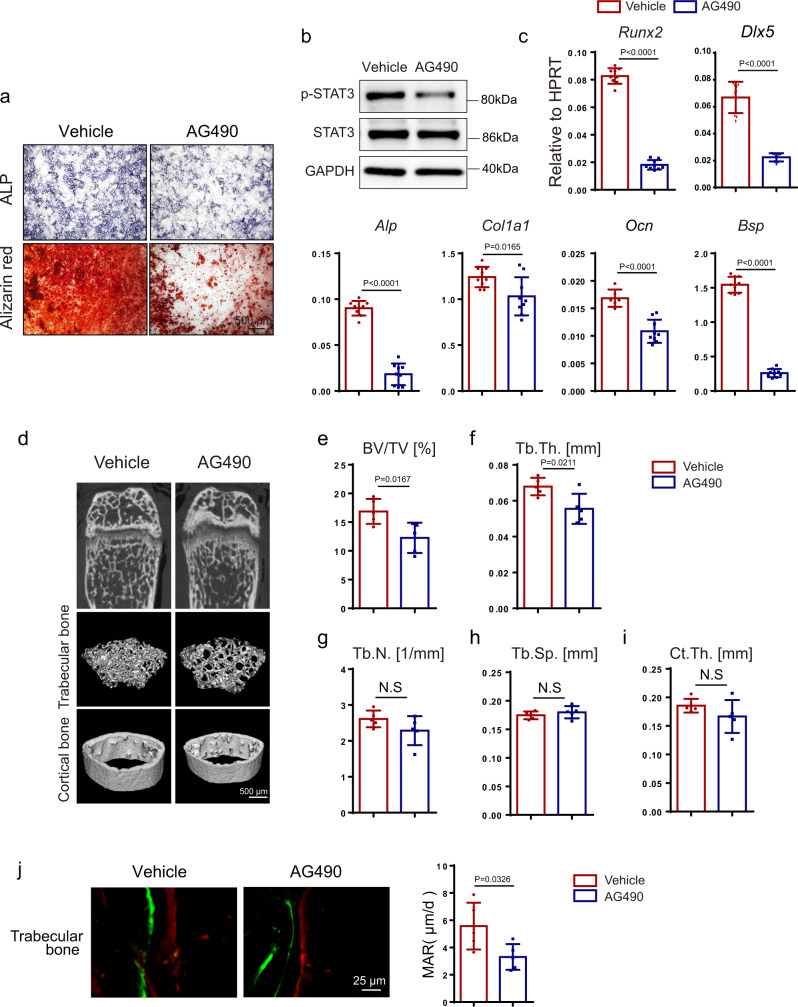
Pharmacological inhibition of STAT3 activity inhibited osteoblast differentiation and induced bone loss by impairing osteogenesis. **a** ALP staining and alizarin red S staining of BMSCs after culture in osteogenic medium with or without AG490 for 7 and 14 days separately. **b** Expression of p-STAT3 and STAT3 in BMSCs with or without AG490 for 12 h analyzed by western blotting. The experiment was performed twice to similar results. **c** The relative mRNA levels of *Runx2, Dlx5, Alp, Col1α1, Ocn*, and *Bsp* in BMSCs with or without AG490 for 14 days were quantified by qPCR. *n* = 9. **d** Micro-CT images of distal femurs from 9-week-old vehicle and AG490-injected mice. Four-week-old male mice were injected with or without 5 mg/kg AG490 daily for 5 weeks. **e**–**i** Quantitative parameters of micro-CT, including BV/TV, Tb.Th., Tb.N., Tb.Sp., and Ct.Th. *n* = 5. **j** Images of calcein-alizarin red S double labeling of femurs from 9-week-old mice with or without AG490 and quantitative parameters of MAR. *n* = 5. Two-tailed Student’s *t* test. Data represent the mean ± s.d. Source data are provided in the Source data file.

The classical JAK2–STAT3 inhibitor, AG490, has been reported to inhibit the phosphorylation and activity of STAT3. First, we confirmed that AG490 could inhibit STAT3 activity in BMSCs, which was indicated by decreased phosphorylation levels of STAT3 at concentrations of 50 μM (Fig. 8b). Next, we found that the osteoblast differentiation of BMSCs compared with the control group could be impaired by AG490, as indicated by remarkably decreased ALP activity and matrix mineralization (Fig. 8a). In addition, the mRNA levels of osteogenic genes such as *Runx2, Dlx5, Col1a1, Ocn*, and *Bsp* were reduced in the AG490 treated groups (Fig. 8c). To further explore the in vivo effects of the pharmacological inhibition of STAT3 activity, we performed intraperitoneal injection of AG490 at a concentration of 5 mg/kg every day in 4-week-old mice for 5 weeks. With micro-CT analysis, we found that trabecular bone was reduced in the AG490 group compared with the control group, indicated by decreased BV/TV and Tb.Th. (Fig. 8d–h), although Ct.Th. was comparable (Fig. 8i, n). Similar to the gene deletion model, AG490 reduced the bone formation (Fig. 8j) and bone resorption in mice (Supplementary Fig. 3b).

**Fig. 9:**
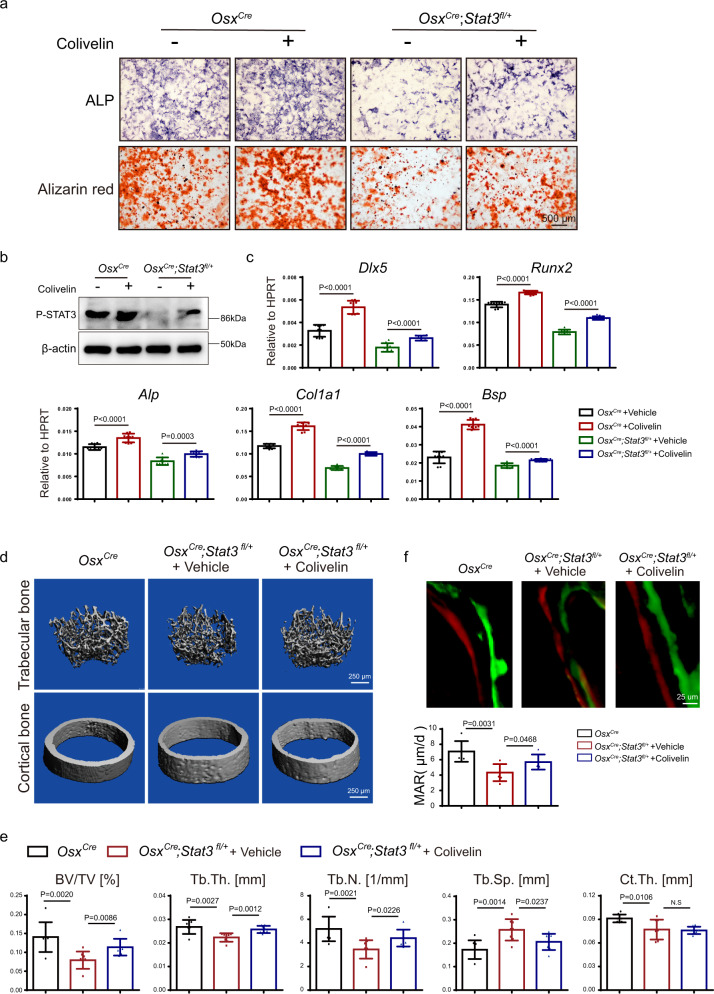
Administration of colivelin promoted osteoblast differentiation and partially rescued bone loss in heterozygous pre-osteoblast-specific *Stat3* deletion mice. **a** ALP and alizarin red S staining of BMSCs from *Osx*^*Cre*^, *Osx*^*Cre*^;*Stat3*^*fl/+*^ mice after culture in osteogenic medium with or without colivelin for 7 and 14 days separately. **b** Expression of p-STAT3 in BMSCs from *Osx*^*Cre*^, *Osx*^*Cre*^;*Stat3*^*fl/+*^ mice treated with or without colivelin for 12 h analyzed by western blotting. The experiment was performed twice to similar results. **c** The relative mRNA levels of *Dlx5*, *Runx2, Alp, Col1α1*, and *Bsp* in BMSCs with or without Colivelin for 14 days were quantified by qPCR. *n* = 9. **d** Micro-CT images of distal femurs from 4-week-old male *Osx*^*Cre*^, *Osx*^*Cre*^;*Stat3*^*fl/+*^+Vehicle, and *Osx*^*Cre*^;*Stat3*^*fl/+*^+ Colivelin group mice. One-week-old male *Osx*^*Cre*^;*Stat3*^*fl/+*^ mice were used to treat with or without 1.5 mg/kg colivelin every day for 3 weeks. **e** Quantitative parameters of micro-CT, including BV/TV, Tb.Th., Tb.N., Tb.Sp., and Ct.Th. *n* = 8. **f** Images of calcein-alizarin red S double labeling of femurs from 4-week-old male *Osx*^*Cre*^, *Osx*^*Cre*^;*Stat3*^*fl/+*^+Vehicle and *Osx*^*Cre*^;*Stat3*^*fl/+*^+ Colivelin group mice and quantitative parameters of MAR. *n* = 6. Two-tailed Student’s *t* test. Data represent the mean ± s.d. Source data are provided in the Source data file.

AD-HIES was caused by heterozygous, and loss-of-function mutation of STAT3, which could induce residual activity and the non-transcriptional functions of STAT3 in the AD-HIES patients^46^. Hence, the heterozygous conditional *Stat3* deletion mice (*Osx*^*Cre*^;*Stat3*^*fl/+*^) may be suitable to functionally mimic the status of *Stat3* mutation in AD-HIES patients and to carry out pharmacological study. We analyzed the skeletal phenotype of the *Stat3* heterozygous mice. The decreased expression of STAT3 in BMSCs from *Osx*^*Cre*^;*Stat3*^*fl/+*^ mice was confirmed by western blotting (Supplementary Fig. 4a). As shown in Supplementary Fig. 4b, heterozygous mice did not show obvious dwarf phenotype. While alcian blue and alizarin red S staining displayed defects of the cranial bones and hypoplasia of the clavicle in the *Osx*^*Cre*^;*Stat3*^*fl/+*^ newborns (Supplementary Fig. 4c–e). Four-week-old *Osx*^*Cre*^;*Stat3*^*fl/+*^ mice exhibited osteoporosis phenotype, which were indicated by reduced BV/TV, Tb.Th., Tb.N., and Ct.Th., as well as increased Tb.Sp. comparing with the control *Osx*^*Cre*^ littermates (Supplementary Fig. 4f–g). Similar to *Osx*^*Cre*^;*Stat3*^*fl/fl*^ mice, the bone formation (Supplementary Fig. 4i) and bone resorption (Supplementary Fig. 4h) decreased in the STAT3 heterozygous mice also. Hence, we raised the question whether pharmacological activation of STAT3 activity by colivelin (MCE, HY-P1061A) could be applied for therapy regarding skeletal deformities in the STAT3 heterozygous mice^47^. Firstly, we isolated BMSCs from 4-week-old *Osx*^*Cre*^;*Stat3*^*fl/+*^ mice and littermate *Osx*^*Cre*^, which were treated with colivelin at concentrations of 1 nM to induce osteogenic differentiation. Western blotting analysis confirmed the increased the phosphorylation level of STAT3 in both colivelin groups in comparison with corresponding control group (Fig. 9b). Colivelin could promote osteoblast differentiation of BMSCs from *Osx*^*Cre*^ as well as rescued the impaired osteoblast differentiation ability of *Osx*^*Cre*^;*Stat3*^*fl/+*^ BMSCs, which was indicated by ALP activity and alizarin red S staining (Fig. 9a). This was consistent with the mRNA level of osteogenic genes, such as *Dlx5*, *Runx2, Alp, Col1a1*, and *Bsp* (Fig. 9c). Next, we treated 1-week-old *Osx*^*Cre*^;*Stat3*^*fl/+*^ mice with colivelin by intraperitoneal administration every other day for 3 weeks. As shown in Fig. 9d, e, the trabecular bone mass decreased of *Osx*^*Cre*^;*Stat3*^*fl/+*^ mice was partially rescued, indicated by increased BV/TV, Tb.Th., Tb.N., and decreased Tb.Sp., while Ct.th. was comparable. Furthermore, calcein-alizarin red S double labeling assay informed that colivelin promoted trabecular bone formation in *Osx*^*Cre*^;*Stat3*^*fl/+*^ mice (Fig. 9f). Colivelin exhibited limited influence on bone resorption, which was demonstrated by comparable numbers of TRAP^+^ osteoclasts (Supplementary Fig. 5a). All the data suggested that colivelin could promote osteoblast differentiation and partially rescued bone loss in heterozygous conditional *Stat3* deletion mice.

**Fig. 10:**
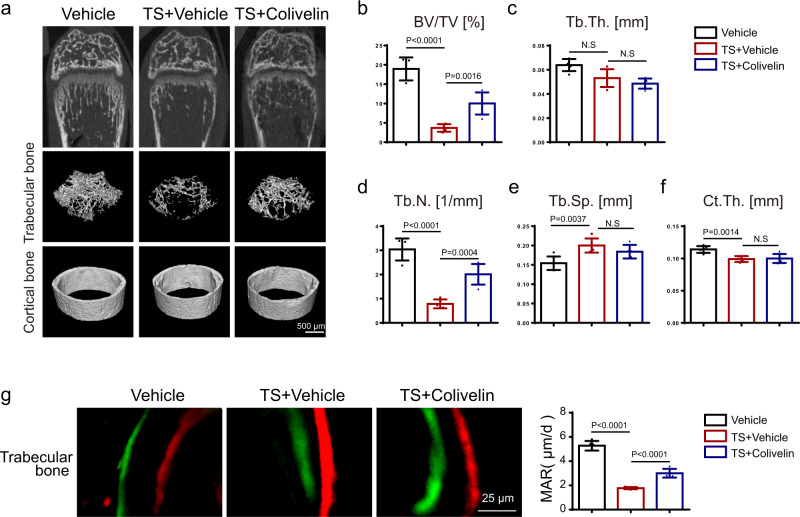
Pharmacological activation of STAT3 activity promoted osteoblast differentiation and partially prevented bone mass loss induced by tail suspension by promoting osteogenesis. **a** Micro-CT images of distal femurs from 5-week-old Vehicle, TS + Vehicle, and TS + Colivelin group mice. Four-week-old male mice were used to perform the tail suspension model with or without 1 mg/kg colivelin every day for 1 week. **b**–**f** Quantitative parameters of micro-CT, including BV/TV, Tb.Th., Tb.N., Tb.Sp., and Ct.Th. *n* = 5. **g** Images of calcein-alizarin red S double labeling of femurs from 5-week-old Vehicle, TS + Vehicle, and TS + Colivelin group mice and quantitative parameters of MAR. *n* = 5. Two-tailed Student’s *t* test. Data represent the mean ± s.d. Source data are provided in the Source data file.

Furthermore, we performed a tail suspension model (TS) to mimic bone loss due to microgravity or disuse^48–50^. This was done in both the presence and absence of colivelin to analyze the effects of colivelin on bone metabolism in vivo. Using micro-CT analysis, we found that tail suspension induced bone loss in the femur as indicated by reduced BV/TV, Tb.N., and Ct.Th. and increased Tb.Sp. (Fig. 10a–f). Bone loss due to tail suspension was partially rescued by colivelin as indicated by increased BV/TV and Tb.N. in the colivelin group (Fig. 10a–f). Further analysis using calcein-alizarin red S double labeling indicated that colivelin promoted bone formation in the tail suspension mice (Fig. 10g), but without any influence on bone resorption as indicated by comparable numbers of TRAP^+^ osteoclasts (Supplementary Fig. 5b).

All these data validated that pharmacological regulation of STAT3 activity could regulate osteoblast differentiation in vitro and modulate bone metabolism in vivo, suggesting that STAT3 may act as a suitable pharmacological target for treatment of AD-HIES-related skeletal defects and other bone disorders by regulating osteogenesis.

## Discussion

Skeletal deformities are typical clinical manifestations of AD-HIES, which are caused mainly by heterozygous, dominant mutations of STAT3^51^. However, the underlying mechanisms remain obscure, which limits the treatment of the AD-HIES-related bone disorders^52,53^. In the present study, we revealed that inactivation of STAT3 in osteoblast lineage cells, but not osteoclasts, induced a series of remarkable bone developmental dysplasia, including dwarfism with short limbs, craniofacial malformations, osteoporosis, and bone fragility, which were similar to the bone defects in AD-HIES. More importantly, these skeletal deformities due to STAT3 deletion in osteoblasts resulted from impaired bone remodeling that was characterized by reduced bone formation. This observation indicated that promoting bone formation, but not inhibiting bone resorption, may be used to treat AD-HIES-related bone disorders such as osteoporosis and recurrent minimal trauma fractures.

Although a series of gene mutations are related to AD-HIES, such as DOCK8, IL-6R, IL6ST, CARD11, gp130, PGM3, ZNF241^5,10–13,54^, AD-HIES is mainly caused by the heterozygous, dominant mutations of STAT3^2^. There were more than 140 *STAT3* gene mutations reported in AD-HIES patients^7,17^. Furthermore, Flanagan et al. revealed that all STAT3 mutations that caused AD-HIES were loss-of-function^55^. We examined the activity of Stat3 gene mutations which were common in AD-HIES patients (R382W, R382Q, V637M, T714A)^7^ and found that these mutations lead to STAT3 loss of function, functionally similar to the effect we observed with *Stat3* knockout model. To further mimic the residual activity induced by the heterozygous, dominant-negative mutation of STAT3 in AD-HIES patients, we analyzed the skeletal phenotype of heterozygous conditional *Stat3* knockout mice. Indeed, as demonstrated in Supplementary Fig. 4, the *Osx*^*Cre*^;*Stat3*^*fl/+*^ mice did not show obvious dwarf phenotype which was the main phenotypic difference between AD-HIES patients and the conditional *Stat3* knockout mice, but exhibited AD-HIES-like skeletal defects, including craniofacial malformation and osteoporosis. These results indicate that *Osx*^*Cre*^;*Stat3*^*fl/+*^ mice were useful for mimicking the skeletal deformity of AD-HIES patients as well as investigating the pharmacological treatment potential of STAT3 activation by colivelin (Fig. 9).

Although early reports suggested an increased bone resorption activity in the AD-HIES patients^27–29^, clinical treatment of patients with bisphosphonates to inhibit osteoclast activity and bone resorption failed to effectively reduce the incidence of pathological fractures in patients in spite of its effect of increased bone mineral density^31,32^. More importantly, in these studies, there were no data examining the correlation between the osteoclast activity change due to STAT3 inactivation and the typical AD-HIES skeletal deformity, such as craniofacial malformation and spontaneous bone fracture. Herein, on one hand, *Stat3* knockout in the mature osteoclast by *Ctsk*^*Cre*^ displayed increased bone mass^34^ rather than any typical AD-HIES skeletal deformities. On the other hand, deletion of *Stat3* either in BMSCs or pre-osteoblasts induced exhibited a series of AD-HIES-like skeletal defects, such as craniofacial malformations, osteoporosis, and bone fragility through impaired osteogenesis as well as reduced osteoblast differentiation. Hence, inactivation of STAT3 in osteoblasts, but not in osteoclasts, induced AD-HIES-related bone deformities.

The head is anatomically the most sophisticated. Craniofacial development depends on the regulation of specific transcription networks and signaling pathways^56^. Transcription factor *Dlx5* plays a critical role in regulating the patterning of craniofacial structures^57,58^. Loss of *Dlx5* resulted in defects in the craniofacial structures. Computed tomography scans showed parietal foramina in the *Osx*^*Cre*^*;Stat3*^*fl/fl*^ mice and morphological deformities including a circular shape and craniosynostosis that were similar to the phenotype of the *Msx1* and *Dlx5* deletion mice. Importantly, RNA-seq revealed that *Dlx5* was significantly downregulated and that overexpression of DLX5 could partially rescue the impaired osteoblast differentiation of the *Stat3*-deficient BMSCs. The activity of the luciferase reporter driven by the *Dlx5* promoter promoted by STAT3 expression and the ChIP assay indicated that STAT3 could bind to the *Dlx5* promoter and regulate the transcription of *Dlx5*. Transcription-regulating *Msx* genes are expressed at multiple sites of tissue-tissue interactions during vertebrate embryonic development^59^. *Msx1* is a key factor in the development of the craniofacial skeleton and has been proven to play an important role in terminal cell differentiation^60^. In addition, previous studies have shown that *Msx1* and *Dlx5* function synergistically to regulate craniofacial development and that *Msx* is crucial for *Dlx5* transcription through binding to its promoter. Here, we have provided evidence that STAT3 cooperated with MSX1 to drive Dlx5 transcription. Co-IP assays revealed that STAT3 physically associated with MSX1. We discovered that STAT3 could further promote MSX1-driven *Dlx5* promoter activity. More importantly, the synergistic effects of STAT3 and MSX1 on *Dlx5* promoter activity were dependent on both the activity of both STAT3 and the STAT3 binding sites on the *Dlx5* promoter. Last, the mutations of highest frequency in STAT3 of AD-HIES could affect the *Dlx5* promoter activity as well as the synergistic effect with MSX1. Taken together, STAT3 may regulate osteoblast differentiation and bone formation by directly promoting osteogenic gene transcription.

The skeleton is not static after being modeled during development, but undergoes lifelong active metabolism called bone remodeling that consists of bone formation by osteoblasts and bone resorption. The balance in bone remodeling is critical for maintaining bone homeostasis, and how to regulate this balance may be the key to the treatment of a series of bone disorders. Here, we have provided evidence that STAT3 in osteoblasts is critical for the regulation of bone metabolism. Firstly, with an inducible osteoblastic *Stat3* deletion mouse, we found that inactivation of STAT3 in osteoblasts impaired bone formation and induced bone loss in adult mice. Moreover, pharmacological inhibition of STAT3 impaired bone formation and induced bone loss in adult mice. Also, pharmacological activation of STAT3 promoted bone formation and reduced bone loss partially due to heterozygous deletion of *Stat3* in pre-osteoblast and tail suspension assay. Hence, we proposed STAT3 to be a suitable pharmacological target for the treatment of AD-HIES-like skeletal defects and other bone metabolism disorders through regulating bone homeostasis.

AD-HIES are mainly caused by heterozygous and dominant-negative mutations in STAT3, resulting in a complicated situation of STAT3 state in patients due to including not only the residual activity of the intact allele but also the dominant-negative functions of the mutant STAT3^17^. There were two germline mouse models of AD-HIES reported previously. One model carried the deletion of SH2 domain of STAT3, displayed early embryonic lethality, and the other model carried a 185 kb bacterial artificial chromosome (BAC) that encoded a common mutation identified in multiple patient cohorts. However, neither of the two models could be employed to study the specific role of STAT3 in bone development and metabolism because of the complexity of whole-body gene mutation. In the current study, we used osteoblast-specific deletion strategy, which we believe would be more appreciate to study STAT3 function in skeletal formation. Although the conditional *Stat3* knockout model could not be genetically resemble the *STAT3* mutations, it could functionally mimic the skeleton defects in patients. There are more than 140 *STAT3* gene mutations reported in AD-HIES patients, with single or combinational mutation^7,17^. In the future, it is promising to establish mouse models with AD-HIES patient-originated *STAT3* mutations or with tissue-specific gain-of-function approach to mimic the authentically status of STAT3 in the patients and reveal their functions in the disease.

In conclusion, our research has clarified a critical role of STAT3 in craniomaxillofacial development and the maintenance of bone homeostasis, as well as revealed its underlying mechanisms. Inactivation of STAT3 in osteoblasts, yet not in osteoclasts, induced AD-HIES-related bone deformities by impairing osteogenesis, which provided potential insights for the treatment of skeletal disorders of AD-HIES patients via the regulation of osteogenesis, though further clinical investigations are needed in the future.

## Methods

### Ethics statement

We have complied with all relevant ethical regulations for animal testing and research. All experimental animal procedures were approved by the Institutional Animal Care and Research Advisory Committee of the Shanghai Ninth People’s Hospital, School of Medicine, Shanghai Jiaotong University and according to the protocol (approval number: HKDL[2018]386) authorized by the Animal Experimental Ethical Inspection Shanghai Ninth People’s Hospital affiliated to shanghai JiaoTong University, School of Medicine.

### Mice

*Stat3*^*fl/fl*^ mice bearing loxP sites flanking exons 18–20 of the *Stat3* gene were purchased from the Jackson Laboratory (No. 016923). *Ctsk*^*Cre*^ strain were provided by S. Kato, University of Tokyo, Tokyo, Japan^61^. *Osx*^*Cre*^ (No. 006361) and *Prx1*^*Cre*^ (No. 005584) strain were purchased from the Jackson Laboratory, and *Col1*^*cre ERT2*^ strain were a gift from Bin Zhou, Shanghai Institutes for Biological Sciences, Chinese Academy of Sciences^62^. All these mice were maintained on the C57BL/6 background. *Stat3*^*fl/fl*^ mice were crossed with *Ctsk*^*Cre*^ mice, *Osx*^*Cre*^ mice, *Prx1*^*Cre*^ mice, or *Col1*^*Cre ERT2*^ mice to generate *Ctsk*^*Cre*^;*Stat3*^*fl/fl*^, *Osx*^*Cre*^;*Stat3*^*fl/+*^, *Osx*^*Cre*^;*Stat3*^*fl/fl*^, *Prx1*^*Cre*^;*Stat3*^*fl/fl*^, or *Col1*^*Cre ERT2*^; *Stat3*^*fl/fl*^ mice. All mice were bred and maintained under specific pathogen-free (SPF) conditions.

### Mice treated with STAT3 inhibitor

Ten 4-week-old male C57BL/6J mice were purchased from Shanghai JSJ Laboratory Animal Co., Ltd., which were randomly divided into two groups. Vehicle group: mice received daily intraperitoneal injections of DPBS (Corning, 21-031-CMR) for 5 weeks. AG490 group: mice received daily intraperitoneal injections of AG490 (5 mg/kg; Selleck, S1143) for 5 weeks. The femurs of the 9-week-old mice were then used for micro-CT or histological analysis.

### Mice treated with STAT3 agonist colivelin

12 one-week-old male *Osx*^*Cre*^;*Stat3*^*fl/+*^ mice were randomly divided into two groups: ‘*Osx*^*Cre*^;*stat3*^*fl/+*^ + Vehicle group’ and ‘*Osx*^*Cre*^;*Stat3*^*fl/+*^ + Colivelin group’.6 littermate *Osx*^*Cre*^ mice were treated as control. In control group, 1-week-old *Osx*^*Cre*^ mice received intraperitoneal administration of PBS every other day for 3 weeks. *Osx*^*Cre*^;*Stat3*^*fl/+*^ + Vehicle group and *Osx*^*Cre*^;*Stat3*^*fl/+*^ + Colivelin group were referred to as 1-week-old *Osx*^*Cre*^;*Stat3*^*fl/+*^ mice that received intraperitoneal administration of PBS and 1.5 mg/kg colivelin (MCE, HY-P1061A), respectively, every other day for 3 weeks. The femurs of the 4-week-old mice were employed for micro-CT and histological analysis.

### Tail suspension model and STAT3 agonist colivelin treatment

15 four-week-old male C57BL/6J mice were purchased from Shanghai JSJ Laboratory Animal Co., Ltd. Which were randomly divided into 3 groups, including ‘Vehicle group’, ‘TS + vehicle group’ and ‘TS + colivelin group’. In Vehicle group, mice received intraperitoneal administration of PBS for 7 days without tail suspension. In TS + vehicle group, mice were suspended in individual plastic cages for 7 days at 30 degrees head-down tilt simultaneously intraperitoneal administration of PBS. The angle of suspension was adjusted to make sure that mouse hind limbs were unable to touch the ground when it was fully stretching^63^. In TS + colivelin group, mice were suspended in individual plastic cages for 7 days and with intraperitoneal administration of 1 mg/kg colivelin (MCE, HY-P1061A) for each day.

### Analysis of bone phenotypes

For alcian blue and alizarin red S staining of the skeleton, briefly, newborn mice were eviscerated and the skin was removed. After fixation with 95% ethanol for 1 day, the newborns were stained for 42 h in alcian blue solution. Then, they were fixed and cleared with 95% ethanol two times, each for 1 h, and then treated with 2% KOH for 3–4 h. After staining with ARS solution for 3–4 h, the skeletons were cleared with 1% KOH in 20% glycerol. For 2-week-old mice, ARS staining needed 6–8 h, and then the skeletons were cleared with 1% KOH in 20% glycerol. For 8-week-old mice, 2% KOH digestion for 6–8 h and ARS staining for 12 h. The skeletons were then cleared with 1% KOH in 20% glycerol.

For histological analysis, bone tissues were fixed in 4% paraformaldehyde (PFA) for 48 h, incubated in 15% DEPC-EDTA for decalcification, and then embedded in paraffin. For embryonic mice, 4-mm tissue sections were used for DIG-labeled in situ hybridization (Roche, No. 11207733910). For postnatal mice, bone tissues were fixed in 4% PFA and decalcified for 2 weeks prior to paraffin embedding. Tissue sections (6 mm) were used for TRAP staining according to the standard protocol (Sigma, 387A-1KT). For safranin O staining, slides were deparaffinized and hydrated to distilled water, then stained with 0.05% fast green (FCF) solution for 1.5 min. Next, they were rinsed quickly with 1% acetic acid solution for no more than 10 s, stained in 0.5% safranin O solution for 1 min, and differentiated with 95% ethanol. Last, they were washed in running water and cleared with xylene. Finally, they were mounted using a resinous medium.

### Immunohistochemical staining and immunofluorescence

Bone tissues were fixed in 4% PFA for 48 h and incubated in 15% EDTA for decalcification. Then, specimens were embedded in paraffin and sectioned at 6 μm. For immunohistochemical staining, sections were de-waxed and rehydrated. A solution of 3% H_2_O_2_ was used to block the activity of endogenous peroxidase. Antigen retrieval was performed with protease K at 37 °C for 15 min. The antibody to DLX5 (Abcam, EPR4488, Rabbit monoclonal, 1:1000) was added and incubated overnight at 4 °C. Anti-rabbit secondary antibody was added and incubated for 1 h at room temperature, followed by color development with an ABC kit (Vector Labs, PK-4000). For immunofluorescence, sections were blocked in PBS with 10% horse serum for 1 h and then stained overnight with anti-OPN (R&D System, #AF808, Goat Polyclonal, 1:200). Donkey anti-goat Cy3 (Absin, abs20028A, Donkey polyclonal, 1:200) was used as the secondary antibody. DAPI (Sigma, D8417) was used for nuclear staining.

### Calcein-alizarin red S labeling

Mice were injected intraperitoneally with 20 mg/kg calcein (Sigma, C0875-5G, 1 mg/ml in 2% NaHCO_3_ solution) and 40 mg/kg alizarin red S (Sigma, A5533-25G, 2 mg/ml in H_2_O) on day 0 and day 4 separately. On day 7, the mice were euthanized, and the bones were fixed and dehydrated and embedded with Embed-812 (Electron Microscopy Sciences). Samples were cut into 5 μm with a hard tissue cutter (RM2265, Leica, Wetzlar, Germany), and fluorescence-labeled images were captured using a microscope (BX51, Olympus).

### Micro-CT analysis

Mouse hind limbs and skulls were harvested, soft tissues were removed, and the remaining tissues were stored in 70% ethanol. The femurs of 4-week-old *Osx*^*Cre*^;*Stat3*^*fl/fl*^ mice and control littermates were scanned with a SkyScan1176 (Bruker, Kartuizersweg, Belgium) at a 8.96 μm resolution for quantitative analysis. The turn on voltage was 65 kV and current density was 300 μA. The region from −40 to −240 slices below the growth plate was analyzed for BV/TV, Tb.Th., Tb.N., and Tb.Sp. The region from −400 to −456 slices below the growth plate was analyzed for Ct.Th. Skulls of 4-week-old *Osx*^*Cre*^;*Stat3*^*fl/fl*^ mice and control littermates were used for Micro-CT analysis (SkyScan 1176, Bruker, Kartuizersweg, Belgium) with 9 μm voxel size. The turn on voltage was 60 kV and current density was 280 μA Micro-CT analysis of the femurs of 6-week-old *Col1*^*cre ERT2*^;*Stat3*^*fl/fl*^ and 9-week-old mice that had been injected with AG490 for 5 weeks, 5-week-old tail-suspended mice injected with colivelin or PBS and their corresponding WT littermates were scanned with a Quantum GX2 (PerkinElmer, Waltham, USA) instrument. The turn on voltage was 80 kV and current density was 88 μA, and 101 slices (9 μm each) immediately below the growth plate in the distal metaphysis of the femur were used for quantification of the bone parameters.

### Cell culture

BMSCs were isolated from the femora and tibiae of 4-week-old mice and cultured in Minimum Essential Medium-α (α-MEM, Corning, 10-022-CVR) containing 10% FBS (Ausbian, WS500T) and 1% Penicillin/Streptomycin (Thermo Fisher Scientific, No. 15140122). We changed half of the culture medium after 3 days of cultivation, and replaced all the culture medium on the 5th day of cultivation, which were counted and plated on the 7th day of culture. For in vitro osteoblast differentiation, 2 × 10^5^ BMSCs were seeded in 12-well plates and cultured in osteogenic medium (Cyagen, MUBMD-90021) and subjected to ALP staining (Beyotime Institute of Biotechnology, P0321S) on day 7 and alizarin red S staining (Cyagen, MUBMD-90021) on day 14.

BMMs were washed out of the femora and tibiae of 4-week-old *Stat3*^*fl/fl*^ and *Ctsk*^*Cre*^;*Stat3*^*fl/fl*^ mice. After centrifugation (900 × g, 3 min), cells were resuspended as a single-cell suspension and cultured in 10 cm dishes with α-MEM containing 10% FBS and 1% Penicillin/Streptomycin for 24 h. Non-adherent cells were collected, centrifuged (900 × *g*, 3 min), and resuspended in α-MEM supplemented with 10 ng/ml M-CSF (Sigma, AF-315-02). Cells were then seeded into 24-well plates with density of 2 × 10^6^ cells/ml. Three days after seeding, BMMs were cultured in osteoclast differentiation medium consisting of α-MEM supplemented with 20 ng/ml M-CSF and 20 ng/ml RANKL (R&D, Minneapolis, MN, 462-TEC-010). The culture medium was replaced after 2 days and every day thereafter until mature multinuclear osteoclasts were formed (5–7 days). Cells were prepared for protein assay.

293T cells were maintained in Dulbecco’s modified Eagle’s medium (DMEM, Corning, 10-013-CVR) containing 10% FBS and 1% Penicillin/Streptomycin. C3H10T1/2 cells were maintained in α-MEM containing 10% FBS and 1% Penicillin/Streptomycin. All the cells were cultured in a 5% CO_2_ humidified incubator at 37 °C.

### Adenoviruses and lentivirus

Cre-EGFP and EGFP adenovirus and DLX5 and EGFP lentivirus were purchased from Hanbio Biotechnology Co. Ltd (Shanghai). For adenoviruses, 2 × 10^5^ BMSCs were seeded in 12-well plates in α-MEM for 24 h, then BMSCs were infected with adenovirus expressing either Cre-EGFP recombinase or EGFP at a multiplicity of infection of 30. After 4–6 h, BMSCs were cultured in osteogenic medium (Cyagen, MUBMD-90021). For lentivirus, 2 × 10^5^ BMSCs were seeded in 12-well plates in α-MEM for 24 h, then BMSCs were infected with lentivirus expressing either DLX5 recombinase or EGFP at a multiplicity of 20. After 24 h, BMSCs were cultured in osteogenic medium (Cyagen, MUBMD-90021).

### Plasmid

The cDNA of STAT3, MSX1, and MSX2 were cloned into a phage-based plasmid. The *Dlx5* promoter was synthesized and cloned into PGL3-based luciferase reporter (DLX5-Luc). The mutant *Dlx5* promoter with deletion of the two predicted STAT3 binding sites (One binding site is in the promoter -980 to -971 region, and one binding site is in the promoter -933 to -918 region) that predicted was synthesized and cloned into PGL3-based luciferase reporter (DLX5-mu-Luc). Constitutively-active STAT3 (STAT3-C) and dominant-negative mutation of STAT3 (STAT3-DN) were provided by Dr. Feng^43^. STAT3 WT(NM_001369512.1), four Hyper-IgE mutants that previously described^3,7^ (R382W, R382Q, V637M, T714A) was synthesized and cloned into pCDH-CMV-MCS-EF1-GFP + Puro (CD513B-1) (NheI/NotI).

### RT-PCR analysis

Total RNA was extracted from osteoclasts using TRIzol (Sigma, T9424) and was reverse-transcribed into cDNA using the Prime Script RT master kit (TakaRa Bio Inc., RR036A). Real-time reverse transcription PCR (RT-PCR) was performed using the Bio-Rad CFX96 system. The primer sets used were as follows:

mouse-*HPRT*-F: GTTAAGCAGTACAGCCCCAAA

mouse-*HPRT*-R: AGGGCATATCCAACAACAAACTT

mouse-*Stat3*-F: CTTGGCCCTTTGGAATGAAG

mouse-*Stat3*-R: CAAGTGAAAGTGACCCCTCC

mouse-*Runx2*-F: CCAACCGAGTCATTTAAG

mouse-*Runx2*-R: GCTCACGTCGCTCATCTTG

mouse-*Osteocalcin*-F: CTTGGTGCACACCTAGCAGA

mouse-*Osteocalcin*-R: CTCCCTCATGTGTTGTCCCT

mouse-*Alp*-F: CGGGACTGGTACTCGGATAA

mouse-*Alp*-R: ATTCCACGTCGGTTCTGTTC

mouse-*Col1a1*-F: GCTCCTCTTAGGGGCCACT

mouse-*Col1a1*-R: CCACGTCTCACCATTGGGG

mouse-*Bsp*-F: CGAAGAAGCAGAAGTGGATG

mouse-*Bsp*-R: GCTTCTTCTCCGTTGTCTCC

mouse-*Dlx5*-F: TCTCTAGGACTGACGCAAACA

mouse-*Dlx5*-R: GTTACACGCCATAGGGTCGC

mouse-*Msx1*-F: TCATGGCCGATCACAGGAAG

mouse-*Msx1*-R: GGAGTCCTCCGACTGAGAAATG.

### RNA-Seq and GO analysis

Total RNA was isolated with TRIzol from the parietal bone of *Osx*^*Cre*^ mice (*n* = 3) and *Osx*^*Cre*^;*Stat3*^*fl/fl*^ mice (*n* = 3) littermates. Complementary DNA library preparation and sequencing were performed according to the Illumina standard protocol. Raw reads were mapped to mm9 using the TopHat version 1.4.1 program. We assigned fragment per kilo base per million (FPKM) as an expression value for each gene using Cufflinks version 1.3.0 software. Then, Cuffdiff software was used to identify differentially expressed genes between *Osx*^*Cre*^;*Stat3*^*fl/fl*^ and control *Osx*^*Cre*^ samples. Differentially expressed gene heat maps were clustered by k-means clustering using the Euclidean distance as the distance and visualized using Java TreeView software. GO analysis was performed with the DAVID online tool. Top GO categories were selected according to the *P* values.

### Western blotting and co-immunoprecipitation

For western blot analysis of parietal bone, total protein was obtained from P0 mice with SDS buffer (TaKaRa, No. 9173). For western blot analysis of BMSCs, total protein was obtained from BMSCs after 7 days of differentiation in osteogenic medium. For western blotting analysis of BMSCs treated with AG490, 5 × 10^5^ BMSCs were seeded in 6-well plates in α-MEM for 48 h and then treated with AG490 for 12 h. Total cells were then used for western blotting. For western blot analysis of BMSCs treated with colivelin, 5 × 10^5^ BMSCs were seeded in 6-well plates in α-MEM for 48 h, then changed to medium supplemented with 1 nM colivelin for 1 h. Total cells were then used for western blotting. Proteins (60 μg of parietal bone protein or 30 μg of cells) were separated by 10% SDS-PAGE followed by western blotting according to the standard protocols. Antibodies used were STAT3 (Santa Cruz, sc-482, Rabbit polyclonal, 1:500), p-STAT3 (CST, #9138, Mouse monoclonal, 1:1000), GAPDH (CST, #2118, Rabbit monoclonal, 1:1000), and β-Actin (CST, #4970, Rabbit monoclonal, 1:1000).

Co-IP was performed according to a method previously described^64^. Briefly, 293T cells were seeded at 6 × 10^6^ cells per 10 cm dish and cultured overnight. Afterward, we transfected 293T cells with Flag-STAT3 with Myc-MSX1 or HA-MSX2, 36–48 h after transfection, the cells were lysed. Whole-cell lysates were used for immunoprecipitation with anti-Flag antibody (Sigma, F3165, Mouse monoclonal, 1:10,000) at 4 °C overnight. Western blot assay was performed with the indicated antibodies (anti-HA, Sigma, H9658, Mouse monoclonal, 1:1000 and anti-Myc, Sigma, SAB4501941, Rabbit polyclonal, 1:2000).

### Chromatin immunoprecipitation (ChIP) and qPCR

ChIP analysis in C3H10T1/2 cells was performed using an enzymatic chromatin immunoprecipitation kit (MILLIPORE, Catalog #17-371) following the instructions of the manufacturer. Briefly, C3H10T1/2 cells at 80–90% confluence in 10 cm dishes were cross-linked with 1% formaldehyde for 10 min at room temperature followed by quenching with glycine. Chromatin digestion was performed to obtain DNA fragments from 250 to 500 bp by micrococcal nuclease (BioruptorTM, Diagenode, Belgium). Immunoprecipitation was performed with STAT3 (CST, #12640, Rabbit monoclonal, 1:50), and IgG (MILLIPORE, No. 12-371B) was used as a negative control. Precipitated DNA was detected by qPCR with specific primers (Fig. 6a). Primers for *Dlx5* promoter F: TGCCTACTTTTCGGTCTTCA; *Dlx5* promoter R: CTCGACTACTTGGAAGCTTC.

### Luciferase reporter assay

We assessed DLX5 or DLX5-mu transcriptional activity via DLX5 or mutant DLX5 responsive Dual-Luciferase Reporter Assay System. 293T cells were plated at a density of 2 × 10^5^ cells/well in a 24-well plate for 18–24 h and transfected by Lipofectamine 2000 (Life Technologies, Inc. 11668019) leveraging luciferase reporter plasmid (DLX5-Luc or DLX5-mu-Luc) and renilla along with STAT3, MSX1, STAT3 WT or various STAT3 mutants (STAT3-C, STAT3-DN, R382W, R382Q, V637M, T714A) according to a previously reported protocol^65^. All wells were supplemented with control empty expression vector plasmids to make the total amount of DNA constant. At 48 h after transfection, the luciferase activity was assayed in cell extract supernatants using the Dual-Luciferase Reporter Assay System according to the manufacturer’s protocol (Promega, Madison, WI. E1910).

### Statistical analysis

Statistical analysis was performed using the GraphPad Prism 6.01 software (GraphPad Software). Cell-based experiments were performed at least twice. Animals were randomized into different groups and at least 3 mice were used for each group, unless otherwise stated. All quantitative data are presented as the mean ± s.d. Student’s *t*-test was used for statistical evaluations of two group comparisons. *P* < 0.05 was considered statistically significant.

### Reporting summary

Further information on research design is available in the Nature Research Reporting Summary linked to this article.

## Supplementary information

Supplementary Information

Reporting Summary

## Data Availability

The RNA sequencing data have been deposited in the Gene Expression Omnibus (GEO) under accession GSE173711. All data and genetic material utilized for this paper are available upon requests to the corresponding author. Source data are provided with this paper.

## Source data

Source Data

## References

[1] Grimbacher B, Holland SM, Puck JM (2005). Hyper-IgE syndromes. Immunological Rev..

[2] Minegishi Y (2007). Dominant-negative mutations in the DNA-binding domain of STAT3 cause hyper-IgE syndrome. Nature.

[3] Holland SM (2007). STAT3 mutations in the hyper-IgE syndrome. N. Engl. J. Med..

[4] Grimbacher B (1999). Hyper-IgE syndrome with recurrent infections—an autosomal dominant multisystem disorder. N. Engl. J. Med..

[5] Schwerd T (2017). A biallelic mutation in IL6ST encoding the GP130 co-receptor causes immunodeficiency and craniosynostosis. J. Exp. Med..

[6] Renner ED (2008). Novel signal transducer and activator of transcription 3 (STAT3) mutations, reduced T(H)17 cell numbers, and variably defective STAT3 phosphorylation in hyper-IgE syndrome. J. Allergy Clin. Immunol..

[7] Woellner C (2010). Mutations in STAT3 and diagnostic guidelines for hyper-IgE syndrome. J. Allergy Clin. Immunol..

[8] Chandesris MO (2012). Autosomal dominant STAT3 deficiency and hyper-IgE syndrome: molecular, cellular, and clinical features from a French national survey. Medicine.

[9] Wu J (2017). Clinical manifestations and genetic analysis of 17 patients with autosomal dominant hyper-IgE syndrome in Mainland China: new reports and a literature review. J. Clin. Immunol..

[10] Spencer S (2019). Loss of the interleukin-6 receptor causes immunodeficiency, atopy, and abnormal inflammatory responses. J. Exp. Med..

[11] Sassi A (2014). Hypomorphic homozygous mutations in phosphoglucomutase 3 (PGM3) impair immunity and increase serum IgE levels. J. Allergy Clin. Immunol..

[12] Béziat, V. et al. A recessive form of hyper-IgE syndrome by disruption of ZNF341-dependent STAT3 transcription and activity. *Sci. Immunol.* **3**, eaat4956 (2018). 10.1126/sciimmunol.aat4956 PMC6141026 29907691

[13] Zhang Q (2009). Combined immunodeficiency associated with DOCK8 mutations. N. Engl. J. Med..

[14] Weber-Nordt RM, Mertelsmann R, Finke J (1998). The JAK-STAT pathway: signal transduction involved in proliferation, differentiation and transformation. Leuk. Lymphoma.

[15] O’Shea JJ, Gadina M, Schreiber RD (2002). Cytokine signaling in 2002: new surprises in the Jak/Stat pathway. Cell.

[16] Johnson DE, O’Keefe RA, Grandis JR (2018). Targeting the IL-6/JAK/STAT3 signalling axis in cancer. Nat. Rev. Clin. Oncol..

[17] Asano, T. et al. Human STAT3 variants underlie autosomal dominant hyper-IgE syndrome by negative dominance. *J. Exp. Med.* **218**, e20202592 (2021). 10.1084/jem.20202592 PMC8217968 34137790

[18] Myles IA (2018). TNF overproduction impairs epithelial staphylococcal response in hyper IgE syndrome. J. Clin. Investig..

[19] Keles S (2016). Dedicator of cytokinesis 8 regulates signal transducer and activator of transcription 3 activation and promotes T(H)17 cell differentiation. J. Allergy Clin. Immunol..

[20] Kreins AY (2015). Human TYK2 deficiency: mycobacterial and viral infections without hyper-IgE syndrome. J. Exp. Med..

[21] Steward-Tharp SM (2014). A mouse model of HIES reveals pro- and anti-inflammatory functions of STAT3. Blood.

[22] Chaudhry A (2009). CD4+ regulatory T cells control TH17 responses in a Stat3-dependent manner. Science.

[23] Zhou H (2011). Osteoblast/osteocyte-specific inactivation of Stat3 decreases load-driven bone formation and accumulates reactive oxygen species. Bone.

[24] Corry KA (2019). Stat3 in osteocytes mediates osteogenic response to loading. Bone Rep..

[25] Ziros PG, Georgakopoulos T, Habeos I, Basdra EK, Papavassiliou AG (2004). Growth hormone attenuates the transcriptional activity of Runx2 by facilitating its physical association with Stat3beta. J. Bone Miner. Res..

[26] Feng X, McDonald JM (2011). Disorders of bone remodeling. Annu. Rev. Pathol..

[27] Cohen-Solal M (1995). Cytokine-mediated bone resorption in patients with the hyperimmunoglobulin E syndrome. Clin. Immunol. Immunopathol..

[28] Zhang Z (2005). Osteoporosis with increased osteoclastogenesis in hematopoietic cell-specific STAT3-deficient mice. Biochem. Biophys. Res. Commun..

[29] Leung DY (1988). Increased in vitro bone resorption by monocytes in the hyper-immunoglobulin E syndrome. J. Immunol..

[30] Staines Boone AT (2016). Zoledronate as effective treatment for minimal trauma fractures in a child with STAT3 deficiency and osteonecrosis of the hip. Pediatr. Blood Cancer.

[31] Sowerwine KJ (2014). Bone density and fractures in autosomal dominant hyper IgE syndrome. J. Clin. Immunol..

[32] Scheuerman O (2013). Reduced bone density in patients with autosomal dominant hyper-IgE syndrome. J. Clin. Immunol..

[33] Dibbs R, Raghuram A, Roy MG, Kaufman MG, Monson LA (2020). Orthognathic Surgical Treatment in a Patient With Hyperimmunoglobulin E Syndrome. J. Craniofacial Surg..

[34] Yang Y (2019). STAT3 controls osteoclast differentiation and bone homeostasis by regulating NFATc1 transcription. J. Biol. Chem..

[35] Davey RA (2012). Decreased body weight in young Osterix-Cre transgenic mice results in delayed cortical bone expansion and accrual. Transgenic Res..

[36] Huang W, Olsen BR (2015). Skeletal defects in Osterix-Cre transgenic mice. Transgenic Res..

[37] Logan M (2002). Expression of Cre Recombinase in the developing mouse limb bud driven by a Prxl enhancer. Genes.

[38] Dirckx N, Moorer MC, Clemens TL, Riddle RC (2019). The role of osteoblasts in energy homeostasis. Nat. Rev. Endocrinol..

[39] Manolagas SC, Jilka RL (1995). Bone marrow, cytokines, and bone remodeling. Emerging insights into the pathophysiology of osteoporosis. N. Engl. J. Med..

[40] Marie PJ (2013). Targeting integrins to promote bone formation and repair. Nat. Rev. Endocrinol..

[41] Artigas N (2017). p53 inhibits SP7/Osterix activity in the transcriptional program of osteoblast differentiation. Cell Death Differ..

[42] Acampora D (1999). Craniofacial, vestibular and bone defects in mice lacking the Distal-less-related gene Dlx5. Development.

[43] Wang G (2016). STAT3 selectively interacts with Smad3 to antagonize TGF-β signalling. Oncogene.

[44] Sugii H (2017). The Dlx5-FGF10 signaling cascade controls cranial neural crest and myoblast interaction during oropharyngeal patterning and development. Development.

[45] Chung IH, Han J, Iwata J, Chai Y (2010). Msx1 and Dlx5 function synergistically to regulate frontal bone development. Genes.

[46] Vogel TP, Milner JD, Cooper MA (2015). The ying and yang of STAT3 in human disease. J. Clin. Immunol..

[47] Wang, X. et al. A potential nutraceutical candidate lactucin inhibits adipogenesis through downregulation of JAK2/STAT3 signaling pathway-mediated mitotic clonal expansion. *Cells* **9**, 331 (2020). 10.3390/cells9020331 PMC7072480 32023857

[48] Aguirre JI (2006). Osteocyte apoptosis is induced by weightlessness in mice and precedes osteoclast recruitment and bone loss. J. Bone Miner. Res..

[49] Kodama Y (1997). Inhibition of bone resorption by pamidronate cannot restore normal gain in cortical bone mass and strength in tail-suspended rapidly growing rats. J. Bone Miner. Res..

[50] Lloyd SA (2014). Interdependence of muscle atrophy and bone loss induced by mechanical unloading. J. Bone Miner. Res..

[51] Béziat, V. et al. Dominant-negative mutations in human IL6ST underlie hyper-IgE syndrome. *J. Exp. Med.* **217**, e20191804 (2020). 10.1084/jem.20191804 PMC7971136 32207811

[52] Dmitrieva NI (2020). Impaired angiogenesis and extracellular matrix metabolism in autosomal-dominant hyper-IgE syndrome. J. Clin. Investig..

[53] Bocchini CE (2016). Protein stabilization improves STAT3 function in autosomal dominant hyper-IgE syndrome. Blood.

[54] Shahin T (2019). Selective loss of function variants in IL6ST cause Hyper-IgE syndrome with distinct impairments of T-cell phenotype and function. Haematologica.

[55] Flanagan SE (2014). Activating germline mutations in STAT3 cause early-onset multi-organ autoimmune disease. Nat. Genet..

[56] Wilkie AO, Morriss-Kay GM (2001). Genetics of craniofacial development and malformation. Nat. Rev. Genet..

[57] Depew MJ (1999). Dlx5 regulates regional development of the branchial arches and sensory capsules. Development.

[58] Han J (2009). Indirect modulation of Shh signaling by Dlx5 affects the oral-nasal patterning of palate and rescues cleft palate in Msx1-null mice. Development.

[59] Alappat S, Zhang ZY, Chen YP (2003). Msx homeobox gene family and craniofacial development. Cell Res..

[60] Blin-Wakkach C (2001). Endogenous Msx1 antisense transcript: in vivo and in vitro evidences, structure, and potential involvement in skeleton development in mammals. Proc. Natl Acad. Sci. USA.

[61] Nakamura T (2007). Estrogen prevents bone loss via estrogen receptor alpha and induction of Fas ligand in osteoclasts. Cell.

[62] He L (2017). Preexisting endothelial cells mediate cardiac neovascularization after injury. J. Clin. Investig..

[63] Chowdhury P, Long A, Harris G, Soulsby ME, Dobretsov M (2013). Animal model of simulated microgravity: a comparative study of hindlimb unloading via tail versus pelvic suspension. Physiological Rep..

[64] Dai Q (2017). mTOR/Raptor signaling is critical for skeletogenesis in mice through the regulation of Runx2 expression. Cell Death Differ..

[65] Liu Z (2016). Mediator MED23 cooperates with RUNX2 to drive osteoblast differentiation and bone development. Nat. Commun..

