# Supplementary material for: STAT3 is critical for skeletal development and bone homeostasis by regulating osteogenesis
Source: Nat Commun. 2021 Nov 25;12:6891. doi: 10.1038/s41467-021-27273-w (PMC8616950; doi:10.1038/s41467-021-27273-w)
Supplement: Supplementary file 1 — Supplementary Information [file 41467_2021_27273_MOESM1_ESM.pdf]

Supplementary Figures

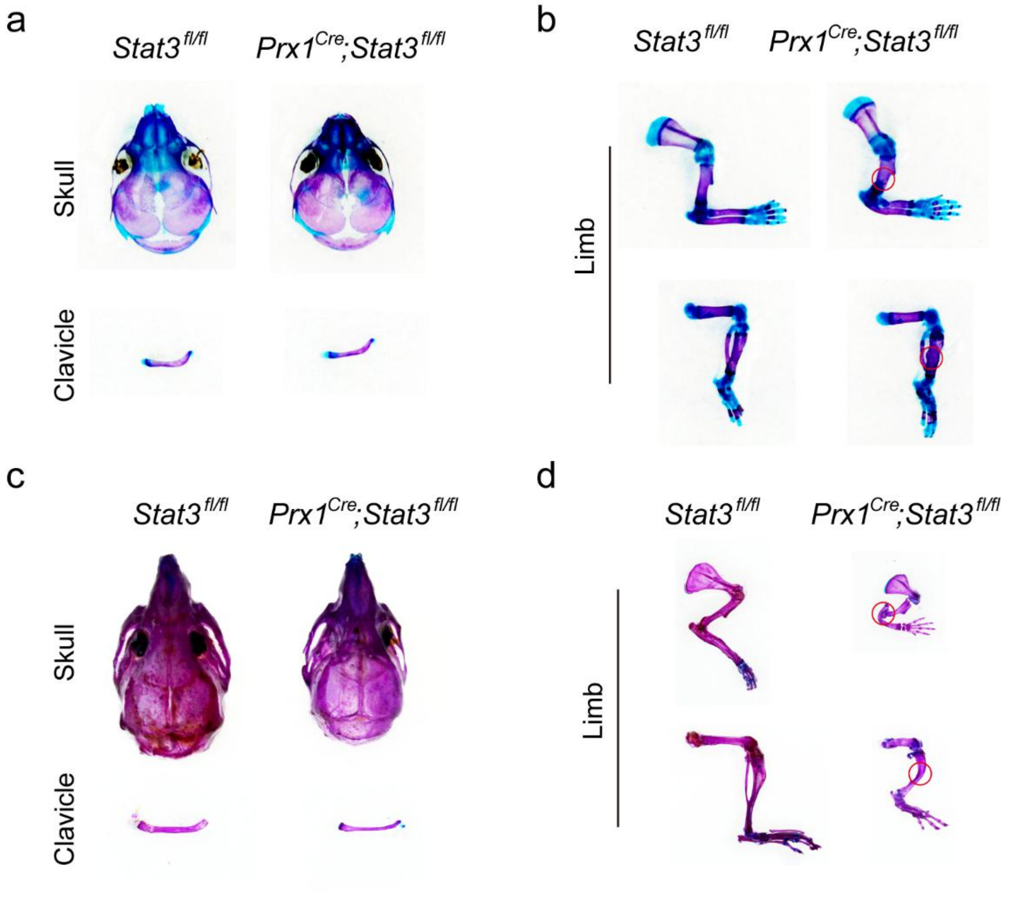

Supplementary Fig.1 **a-b** Skeletal preparations from male *Stat3*$^{fl/fl}$ and *Prx1*^{Cre}*;Stat3*$^{fl/fl}$ newborns were double-stained with alcian blue and alizarin red S. **c-d** Skeletal preparations from 8-week-old male *Stat3*$^{fl/fl}$ and *Prx1*^{Cre}*;Stat3*$^{fl/fl}$ mice were double-stained with alcian blue and alizarin red S. Red circles represent bone fracture.

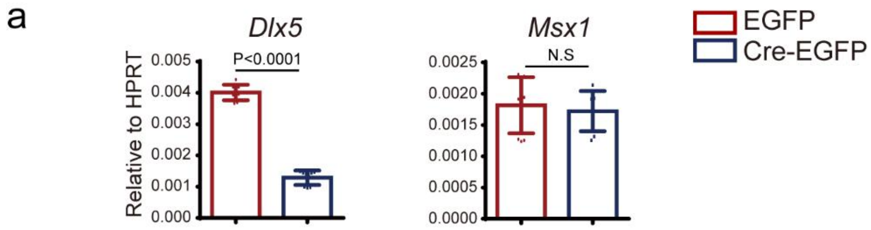

Supplementary Fig.2 **a** BMSCs from 4-week-old male *Stat3*$^{fl/fl}$ mice were infected with adenovirus expressing EGFP or Cre-EGFP for 4hours then induced by osteogenic medium, the relative mRNA levels of *Dlx5* and *Msx1* were quantified after 14 days of culture in osteogenic medium. n=9. Two-tailed Student's t test. Data represent the mean ± s.d. Source data are provided in the Source Data File.

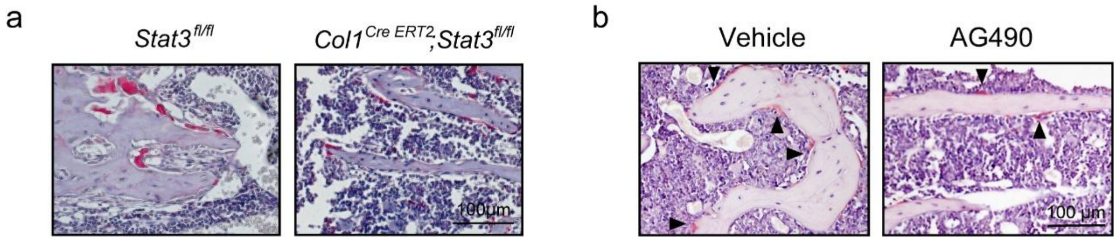

Supplementary Fig.3 **a** TRAP staining of femurs from 6-week-old male *Stat3*$^{fl/fl}$ and *Col1*$^{Cre\ ERT2}$; *Stat3*$^{fl/fl}$ mice. **b** TRAP staining of femurs from 9-week-old male mice with or without AG490. Black triangles indicate TRAP^{+}- multinucleated osteoclasts. The TRAP staining were repeated twice to similar results.

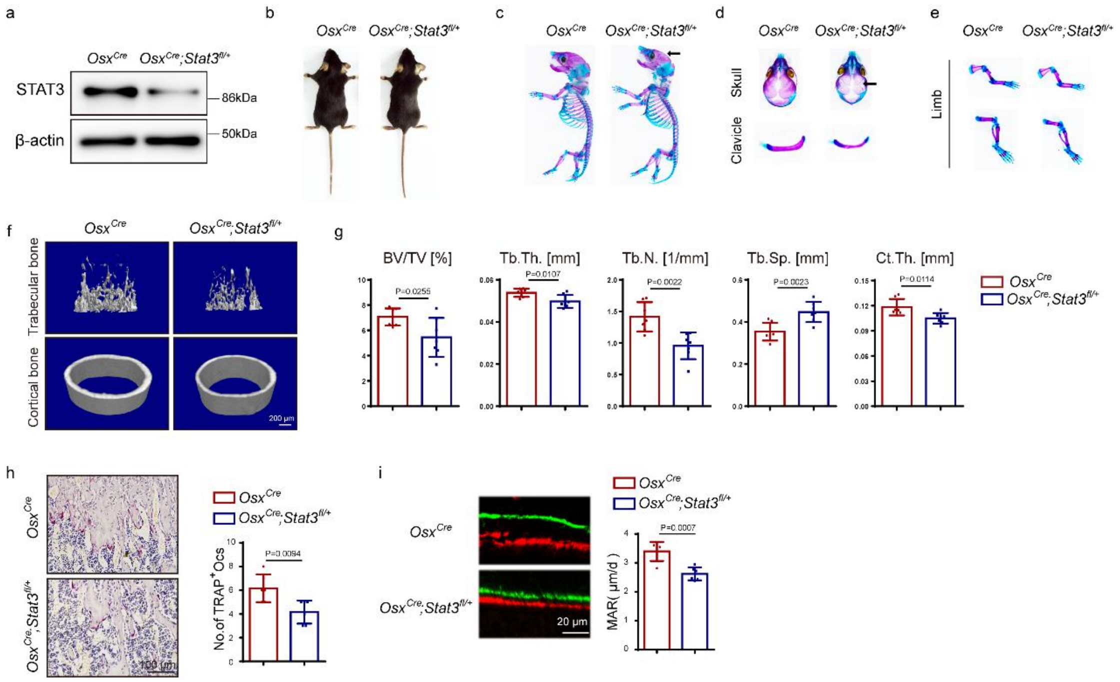

Supplementary Fig.4 **Heterozygous mutation in the STAT3 gene induced skeletal deformities. a** Expression of STAT3 in BMSCs from 4-week-old male *Osx*^{Cre} and *Osx*^{Cre}*;Stat3*$^{fl/+}$ mice cultured in osteogenic medium for 7 days. The experiment was performed twice to similar results. **b** Representative views of 8-week-old male *Osx*^{Cre} and *Osx*^{Cre}*;Stat3*$^{fl/+}$ mice. **c-e** Skeletal preparations from male *Osx*^{Cre} and *Osx*^{Cre}*;Stat3*$^{fl/+}$ newborns were double-stained with alcian blue and alizarin red S. Arrows indicate calvarial lesions. **f** Micro-CT images of distal femurs from 4-week-old male *Osx*^{Cre} and *Osx*^{Cre}*;Stat3*$^{fl/+}$ mice. **g** Quantitative parameters of micro-CT, including BV/TV, tTb.Th., Tb.N., Tb.Sp., and Ct.Th.. n=7. **h** TRAP staining of femurs from 4-week-old male *Osx*^{Cre} and *Osx*^{Cre}*;Stat3*$^{fl/+}$ mice and analysis of the number of TRAP^{+}-multinucleated osteoclasts. n=6. **i** Representative images of calcein-alizarin red S double labeling of femurs from 4-week-old male *Osx*^{Cre} and *Osx*^{Cre}*;Stat3*$^{fl/+}$ mice and quantitative parameters mineral apposition rate (MAR). n=6. Two-tailed Student's t test. Data represent the mean ± s.d. Source data are provided in the Source Data File.

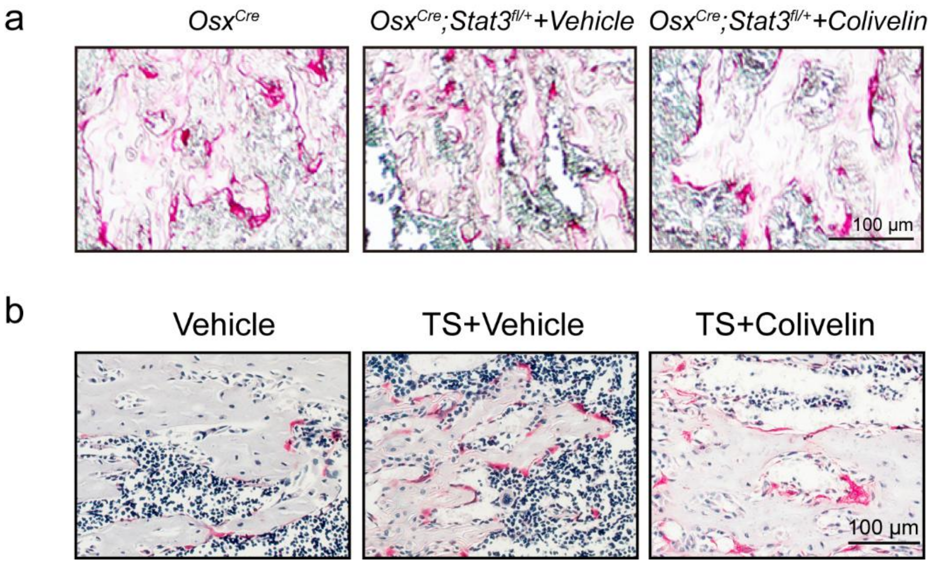

Supplementary Fig.5 **a** TRAP staining of femurs from 4-week-old male *Osx*^{Cre}, *Osx*^{Cre};S*tat3*$^{fl/+}$+Vehicle and *Osx*^{Cre};S*tat3*$^{fl/+}$+ Colivelin group mice. **b** TRAP staining of femurs from 5-week-old male Vehicle, TS+Vehicle and TS+Colivelin group mice.The TRAP staining were repeated twice to similar results.

Supplementary Table 1

	Antibodies	Sources	Cat. No.	Clone Name	Dilutions	Applications
1	Anti-Dlx5	Abcam	EPR4488	Rabbit monoclonal	1:1000	IHC
2	Anti-OPN	R&D System	#AF808	Goat polyclonal	1:200	IF
3	Anti-Stat3	Santa Cruz	sc-482	Rabbit polyclonal	1:500	WB
4	Anti-pStat3	CST	#9138	Mouse monoclonal	1:1000	WB
5	Anti-GAPDH	CST	#2118	Rabbit monoclonal	1:1000	WB
6	Anti-β-Actin	CST	#4970	Rabbit monoclonal	1:1000	WB
7	Anti-FLAG	Sigma	F3165	Mouse monoclonal	1:10000	Co-IP
8	Anti-HA	Sigma	H9658	Mouse monoclonal	1:1000	Co-IP
9	Anti-MYC	Sigma	SAB4501941	Rabbit polyclonal	1:2000	Co-IP
10	Anti-Cy3	Absin	abs20028A	Donkey polyclonal	1:200	IF
11	Anti-Stat3	CST	#12640	Rabbit monoclonal	1:50	CHIP

Supplementary Table 2

S.No.	Gene name (Mus musculus)	Direction	Sequence
1	*HPRT*	Forward primer	5′-GTTAAGCAGTACAGCCCCAAA-3'
		Reverse primer	5′-AGGGCATATCCAACAACAAACTT-3'
2	*Stat3*	Forward primer	5′-CTTGGCCCTTTGGAATGAAG-3'
		Reverse primer	5′-CAAGTGAAAGTGACCCCTCC-3'
3	*Runx2*	Forward primer	5′-CCAACCGAGTCATTTAAG-3'
		Reverse primer	5′-GCTCACGTCGCTCATCTTG-3'
4	*Osteocalcin*	Forward primer	5′-CTTGGTGCACACCTAGCAGA-3'
		Reverse primer	5′-CTCCCTCATGTGTTGTCCCT-3'
5	*Alp*	Forward primer	5′- CGGGACTGGTACTCGGATAA-3'
		Reverse primer	5′-ATTCCACGTCGGTTCTGTTC-3'
6	*Col1a1*	Forward primer	5′-GCTCCTCTTAGGGGCCACT-3'
		Reverse primer	5′-CCACGTCTCACCATTGGGG-3'
7	*Bsp*	Forward primer	5′-CGAAGAAGCAGAAGTGGATG-3'
		Reverse primer	5′-GCTTCTTCTCCGTTGTCTCC-3'
8	*Dlx5*	Forward primer	5′-TCTCTAGGACTGACGCAAACA-3'
		Reverse primer	5′-GTTACACGCCATAGGGTCGC-3'
9	Msx1	Forward primer	5′-TCATGGCCGATCACAGGAAG-3'
		Reverse primer	5′-GGAGTCCTCCGACTGAGAAATG-3'
10	*Dlx5*-Chip	Forward primer	5′-TGCCTACTTTTCGGTCTTCA-3'
		Reverse primer	5′-CTCGACTACTTGGAAGCTTC-3'
