# Supplementary material for: STAT3 is critical for skeletal development and bone homeostasis by regulating osteogenesis
Source: Nat Commun. 2021 Nov 25;12:6891. doi: 10.1038/s41467-021-27273-w (PMC8616950; doi:10.1038/s41467-021-27273-w)
Supplement: Supplementary file 2 — Reporting Summary [file 41467_2021_27273_MOESM2_ESM.pdf]

nature portfolio

Corresponding author(s): Lingyong Jiang

Last updated by author(s): Oct 9, 2021

Reporting Summary

Nature Portfolio wishes to improve the reproducibility of the work that we publish. This form provides structure for consistency and transparency in reporting. For further information on Nature Portfolio policies, see our Editorial Policies and the Editorial Policy Checklist.

Statistics

For all statistical analyses, confirm that the following items are present in the figure legend, table legend, main text, or Methods section.

n/a	Confirmed	
☐	☒	The exact sample size (*n*) for each experimental group/condition, given as a discrete number and unit of measurement
☐	☒	A statement on whether measurements were taken from distinct samples or whether the same sample was measured repeatedly
☐	☒	The statistical test(s) used AND whether they are one- or two-sided<br>*Only common tests should be described solely by name; describe more complex techniques in the Methods section.*
☒	☐	A description of all covariates tested
☒	☐	A description of any assumptions or corrections, such as tests of normality and adjustment for multiple comparisons
☐	☒	A full description of the statistical parameters including central tendency (e.g. means) or other basic estimates (e.g. regression coefficient) AND variation (e.g. standard deviation) or associated estimates of uncertainty (e.g. confidence intervals)
☐	☒	For null hypothesis testing, the test statistic (e.g. *F*, *t*, *r*) with confidence intervals, effect sizes, degrees of freedom and *P* value noted<br>*Give P values as exact values whenever suitable.*
☒	☐	For Bayesian analysis, information on the choice of priors and Markov chain Monte Carlo settings
☒	☐	For hierarchical and complex designs, identification of the appropriate level for tests and full reporting of outcomes
☒	☐	Estimates of effect sizes (e.g. Cohen's *d*, Pearson's *r*), indicating how they were calculated

Our web collection on statistics for biologists contains articles on many of the points above.

Software and code

Policy information about availability of computer code

Data collection	Fluorescence-labeled images were captured using a microscope (BX51, Olympus). Real-time reverse transcription PCR (RT-PCR) was performed using the Bio-Rad CFX96 system. The femurs of mice were scanned with a SkyScan1176 (Bruker, Kartuizersweg, Belgium) and Quantum GX2（PerkinElmer, Waltham, USA） instrument.
Data analysis	Statistical analysis was performed using GraphPad Prism 6.01 software.The quantification of μ-CT was performed using Micro-CT (SkyScan 1176, Bruker, Kartuizersweg, Belgium) or Quantum GX2（PerkinElmer, Waltham, USA） instrument analysis. RNA-seq analysis was performed with opHat version 1.4.1 program and Cufflinks version 1.3.0 software. Differentially expressed gene heat maps visualized using Java TreeView software. GO analysis was performed with the DAVID online tool.

For manuscripts utilizing custom algorithms or software that are central to the research but not yet described in published literature, software must be made available to editors and reviewers. We strongly encourage code deposition in a community repository (e.g. GitHub). See the Nature Portfolio guidelines for submitting code & software for further information.

Data

Policy information about availability of data

All manuscripts must include a data availability statement. This statement should provide the following information, where applicable:

- Accession codes, unique identifiers, or web links for publicly available datasets
- A description of any restrictions on data availability
- For clinical datasets or third party data, please ensure that the statement adheres to our policy

Source data for Fig.1-10 and Supplementary Figure 2,4 are provided in the Source Data File. The RNA sequencing data have been deposited in the Gene Expression Omnibus (GEO) under accession GSE173711. All data and genetic material utilized for this paper are available upon requests to corresponding author.

Field-specific reporting

Please select the one below that is the best fit for your research. If you are not sure, read the appropriate sections before making your selection.

- [x] Life sciences
- [ ] Behavioural & social sciences
- [ ] Ecological, evolutionary & environmental sciences

For a reference copy of the document with all sections, see nature.com/documents/nr-reporting-summary-flat.pdf

Life sciences study design

All studies must disclose on these points even when the disclosure is negative.

Sample size	Generally, sample sizes were calculated on the assumption that a 30% difference in the parameters measured would be considered biologically significant with an estimate of sigma of 10-20% of the expected mean. Alpha and Beta were set to the standard values of .05 and 0.2, respectively.
Data exclusions	No data were excluded in this study.
Replication	All experiments reported in the manuscript were replicated successfully to confirm reproducibility.<br>1) Immunoblotting on samples included at least 2 independent experiments, each with consistent results.<br>2) Animal studies were reproduced across at least 3 independent cohorts.<br>3) RT-PCR on mouse bone included at least 9 independent samples per each genotype and experimental group,respectively.<br>4) Alcian blue and alizarin red S staining and RNA in situ on mouse samples included at least 2 independent experiments.<br>5) Cell-based experiments were performed at least twice.
Randomization	Samples and mice were randomly allocated to different groups. Mice used in this study were age- and sex-matched in the same experiment.
Blinding	Mice were blinded to group allocation.

Reporting for specific materials, systems and methods

We require information from authors about some types of materials, experimental systems and methods used in many studies. Here, indicate whether each material, system or method listed is relevant to your study. If you are not sure if a list item applies to your research, read the appropriate section before selecting a response.

Materials & experimental systems

n/a	Involved in the study	
☐	☒	Antibodies
☐	☒	Eukaryotic cell lines
☒	☐	Palaeontology and archaeology
☐	☒	Animals and other organisms
☒	☐	Human research participants
☒	☐	Clinical data
☒	☐	Dual use research of concern

Methods

n/a	Involved in the study	
☒	☐	ChIP-seq
☒	☐	Flow cytometry
☒	☐	MRI-based neuroimaging

Antibodies

Antibodies used	Primary antibodies used for immunoblotting , Co-IP, iImmunohistochemical , imunofluorescence staining and CHIP were specific for anti-Dlx5(Abcam,EPR4488,Rabbit monoclonal,GR225395-14,1:1000), anti-OPN(R&D System,#AF808,Goat Polyclonal, BDO0618021, 1:200),anti-Stat3(Santa Cruz,sc-482 Rabbit polyclonal, A1816,1:500),anti-pStat3(CST,#9138, Mouse monoclonal, 9, 1:1000), anti-GAPDH (CST, #2118, Rabbit monoclonal, 14, 1:1000), anti-β-Actin (CST, #4970, Rabbit monoclonal ,15, 1:1000), anti-FLAG (Sigma, F3165, Mouse monoclonal, SLCC4005, 1:10000), anti-HA(Sigma, H9658, Mouse monoclonal ,128M4789V, 1:1000),anti-MYC (Sigma ,SAB4501941, Rabbit polyclonal, 3110262, 1:2000) ,anti-Cy3 (Absin , abs20028A, Donkey polyclonal, NW07,1:200),and anti-Stat3 (CST,#12640 ,Rabbit monoclonal, 4 ,1:50)
Validation	The following validation method was conducted for IF and IHC usinganti-Dlx5(Abcam, EPR4488,Rabbit monoclonal, GR225395-14, 1:1000), anti-OPN(R&D System,#AF808,Goat Polyclonal, BDO0618021, 1:200): secondary antibody was added alone without primary antibody addition as negative control. The validation statements of the other antibodies can be found on the manufacturer's website.

Eukaryotic cell lines

Policy information about cell lines

Cell line source(s)	C3H10T1/2 (ATCC, CRL-3268); HEK293T (Catalogue number SCSP-502) cell line was ordered from the cell bank of the Chinese

	Academy of Sciences
Authentication	The cell line has been validated using the short tandem repeat (STR) profiling method by the cell bank of Chinese Academy of Sciences.
Mycoplasma contamination	Not tested.
Commonly misidentified lines (See ICLAC register)	No commonly misidentified cell lines were used.

Animals and other organisms

Policy information about studies involving animals; ARRIVE guidelines recommended for reporting animal research

Laboratory animals	Stat3fl/fl mice bearing loxP sites flanking exons 18–20 of the Stat3 gene were purchased from the Jackson Laboratory (No.016923). CtskCre strain were provided by S. Kato, University of Tokyo, Tokyo, Japan59. OsxCre (No.006361) and Prx1Cre (No.005584) strain were purchased from the Jackson Laboratory, and Col1cre ERT2 strain were a gift from Bin Zhou, Shanghai Institutes for Biological Sciences, Chinese Academy of Sciences. Stat3fl/fl mice were crossed with CtskCre mice, OsxCre mice, Prx1Cre mice, or Col1Cre ERT2 mice to generate CtskCre;Stat3fl/fl , OsxCre;Stat3fl/+ , OsxCre;Stat3fl/fl , Prx1Cre;Stat3fl/fl , or Col1Cre ERT2; Stat3fl/fl mice. 4-week-old male OsxCre;Stat3fl/fl mice and OsxCre;Stat3fl/+ mice ,6-week-old male Col1Cre ERT2 mice,9-week-old male AG490 injected mice, 4-week-old male OsxCre;Stat3fl/++ Colivelin group mice, 5-week-old TS+Colivelin group mice with corresponding WT or Vehicle group mice were used for uCT analysis. Both female and male newborns were used for alcian blue and alizarin red S staining. BMSCs were isolated from the 4-week-old mice and induced osteoblast differentiation.<br>All these mice were maintained on the C57BL/6 background. Mice were maintained in a controlled environment with 12:12 light-dark cycle,room temperature of 20.5-22.5℃ and humidity of 30-70%, and had ad libitum access to dry laboratory food and water.
Wild animals	Our study did not involve wild animals.
Field-collected samples	The study did not involve samples collected from the field.
Ethics oversight	All experimental animal procedures were approved by the Institutional Animal Care and Research Advisory Committee of the Shanghai Ninth People's Hospital, School of Medicine, Shanghai Jiaotong University and according to the protocol (approval number: HKDL[2018]386) authorized by the Animal Experimental Ethical Inspection Shanghai Ninth People's Hospital affiliated to shanghai JiaoTong University, School of Medicine.

Note that full information on the approval of the study protocol must also be provided in the manuscript.
