# Supplementary figures and images for: STAT3 is critical for skeletal development and bone homeostasis by regulating osteogenesis
Source: Nat Commun. 2021 Nov 25;12:6891. doi: 10.1038/s41467-021-27273-w (PMC8616950; doi:10.1038/s41467-021-27273-w)

Figure 1B

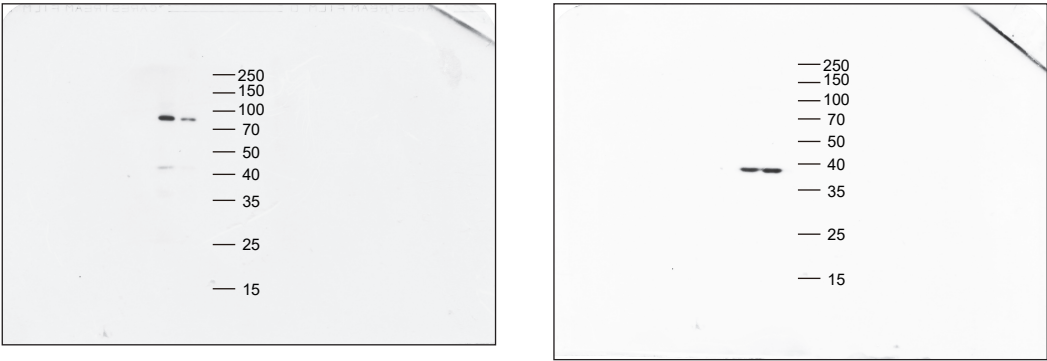

Figure 2B、3B

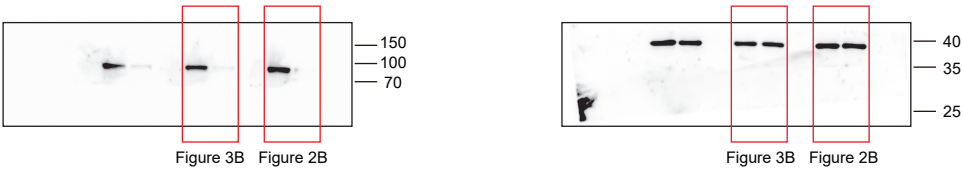

Figure 6E

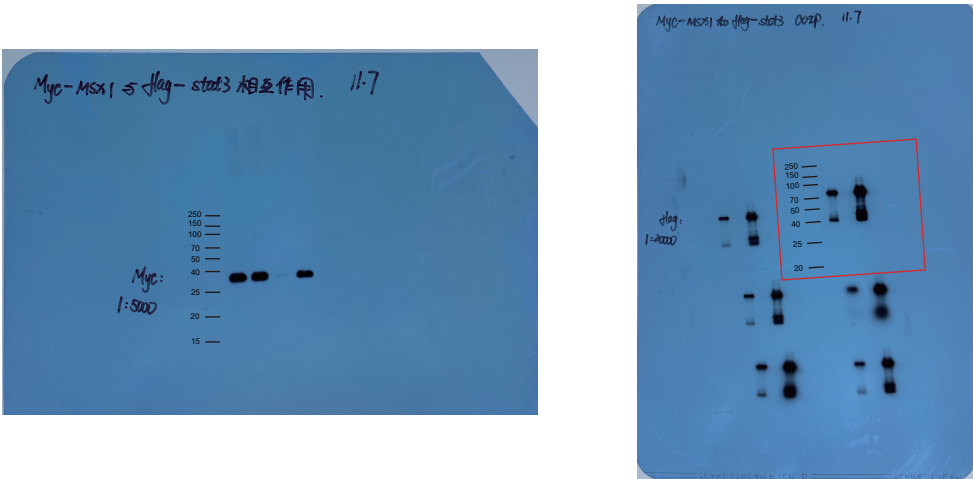

Figure 6F

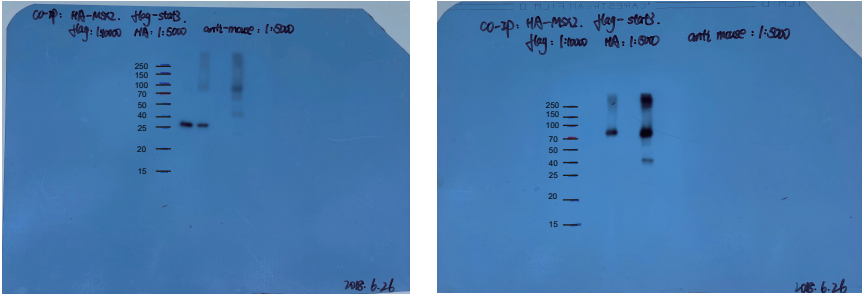

Figure 8B

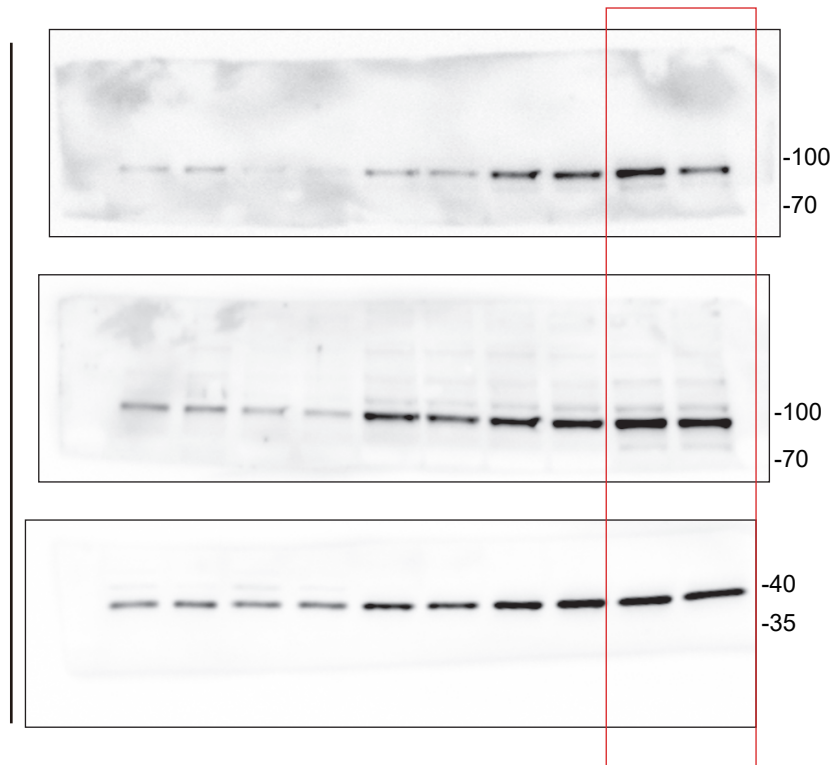

Figure 9B

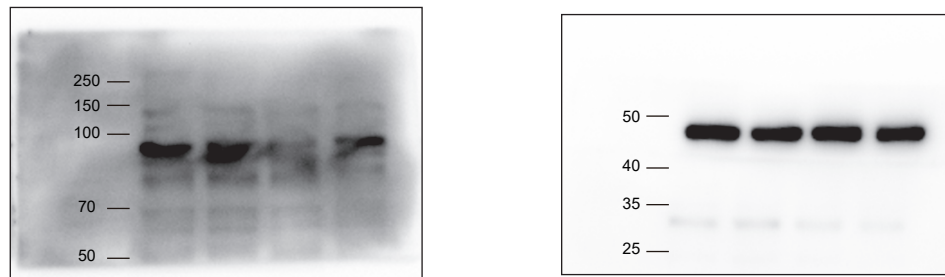

Figure S4A

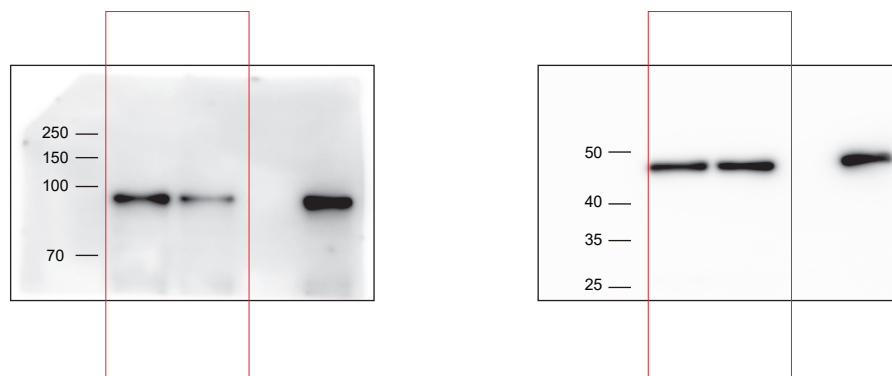

Supplement: Supplementary file 3 — Source Data [file 41467_2021_27273_MOESM3_ESM.zip › Source Data/Western blotting-Source data.pdf]
